# CRISPR screening of porcine sgRNA library identifies host factors associated with Japanese encephalitis virus replication

**DOI:** 10.1038/s41467-020-18936-1

**Published:** 2020-10-14

**Authors:** Changzhi Zhao, Hailong Liu, Tianhe Xiao, Zichang Wang, Xiongwei Nie, Xinyun Li, Ping Qian, Liuxing Qin, Xiaosong Han, Jinfu Zhang, Jinxue Ruan, Mengjin Zhu, Yi-Liang Miao, Bo Zuo, Kui Yang, Shengsong Xie, Shuhong Zhao

**Affiliations:** 1 Key Laboratory of Agricultural Animal Genetics, Breeding and Reproduction of Ministry of Education & Key Lab of Swine Genetics and Breeding of Ministry of Agriculture and Rural Affairs, Huazhong Agricultural University, 430070 Wuhan, P. R. China; 2 The Cooperative Innovation Center for Sustainable Pig Production, Huazhong Agricultural University, 430070 Wuhan, P. R. China; 3 State Key Laboratory of Agriculture Microbiology, Huazhong Agricultural University, 430070 Wuhan, P. R. China; 4 Louisiana State University, School of Veterinary Medicine, Baton Rouge, LA 70803 USA

**Keywords:** High-throughput screening, Animal breeding

## Abstract

Japanese encephalitis virus (JEV) is a mosquito-borne zoonotic flavivirus that causes encephalitis and reproductive disorders in mammalian species. However, the host factors critical for its entry, replication, and assembly are poorly understood. Here, we design a porcine genome-scale CRISPR/Cas9 knockout (PigGeCKO) library containing 85,674 single guide RNAs targeting 17,743 protein-coding genes, 11,053 long ncRNAs, and 551 microRNAs. Subsequently, we use the PigGeCKO library to identify key host factors facilitating JEV infection in porcine cells. Several previously unreported genes required for JEV infection are highly enriched post-JEV selection. We conduct follow-up studies to verify the dependency of JEV on these genes, and identify functional contributions for six of the many candidate JEV-related host genes, including *EMC3* and *CALR*. Additionally, we identify that four genes associated with heparan sulfate proteoglycans (HSPGs) metabolism, specifically those responsible for HSPGs sulfurylation, facilitate JEV entry into porcine cells. Thus, beyond our development of the largest CRISPR-based functional genomic screening platform for pig research to date, this study identifies multiple potentially vulnerable targets for the development of medical and breeding technologies to treat and prevent diseases caused by JEV.

## Abstract (web summary)

Here the authors report the construction of a genome-scale porcine CRISPR/Cas9 library, called PigGeCKO, for screening and analyses of host resistance genes and factors associated with Japanese encephalitis virus replication.

## Introduction

Numerous viruses in nature are capable of infecting both humans and domestic animals. Among these is Japanese encephalitis virus (JEV), a flavivirus which is closely related to Dengue Virus (DENV), Zika Virus (ZIKV), Yellow Fever Virus (YFV), West Nile Virus (WNV), and Hepatitis C Virus (HCV)^1,2^. JEV is the leading cause of viral encephalitis in humans in some Asian countries, with an estimated 69,000 severe clinical cases and ~13,600–20,400 deaths annually^3^, despite the widespread use of vaccine. While several inactivated and live vaccines are used to prevent JEV infection, no antiviral drugs are available for the treatment of JEV-related diseases^4,5^. Despite achievements in control and prevention of JEV infection, this disease remains a major public health concern in Northern Oceania and in South, East, and Southeast Asia, and it’s viewed as an emerging global pathogen^6^.

JEV is a mosquito-borne virus that can be greatly amplified in pigs, causing encephalitis and reproductive complications in swine species. Pigs are readily infected with JEV and can develop high levels of viremia^7,8^. JEV infection is usually asymptomatic in adult pigs, but manifestations of abortion, still-birth, and birth defects, including central nervous system defects, are not unusual following infection in pregnant swine, which can result in substantial economic losses to pork producers^9,10^. Accordingly, JEV outbreaks represent a major threat to both public health and the agricultural economy, especially in areas with low vaccine coverage and/or limited diagnostic capacity^11^. Currently, JEV is only endemic in the Asia-Pacific region; however, it was previously shown that domesticated pigs could potentially become amplification hosts upon the introduction of JEV in other countries^12^.

The JEV infection cycle starts with binding to unknown cellular receptors and attachment factors^6,13^, followed by viral entry to enable replication. Subsequently, the JEV RNA genome is replicated, viral particles are matured, packaged, and released from cells. JEV infects a variety of cell types from diverse species (including mammals, birds, amphibians, and insects), suggesting that JEV can likely access multiple cell types using multiple host receptors^14^. In recent years, tremendous progress has been made in understanding the viral components required for the various steps of JEV entry and replication, but little is known about the host cell components involved in this process^15–18^. Understanding virus–host interactions by elucidating the molecular mechanisms of viral transmission can help identify potential antiviral targets for developing both prophylactic and therapeutic medicines.

Efforts to treat and prevent viral infections have traditionally been aided by genetic screening research to improve our understanding of viral dependencies and to identify potential antiviral strategies. The emergence of CRISPR genetic screening tools has spurred a new era of efficient, versatile, and large-scale screening efforts, with notable examples for flaviviridae family viruses^19–21^. Indeed, work in human cell lines based on CRISPR-based screening strategies (with virus-induced cell death readout phenotypes) have successfully identified required host genes for infection by DENV, ZIKV, WNV, YFV, and HCV^20–24^. These studies have repeatedly illustrated that genome-scale CRISPR screening represents a powerful tool for both basic biology and medical research. However, we are unaware of any genome-scale efforts to examine JEV infection; moreover, there are no reports of genome-scale CRISPR/Cas9 libraries for screening studies in pigs.

Aiming to develop such a resource, and specifically seeking to study the genetic basis of resistance against JEV infection, we develop a resource which we term PigGeCKO (for porcine genome-scale CRISPR knockout), which is comprised of a library of ~85,000 sgRNA constructs (both as plasmids and as prepared lentiviruses). After developing this genome-scale sgRNA library, we infect JEV-susceptible porcine kidney-15 (PK-15) cells^25^ stably expressing the Cas9 protein with the pooled lentiviral sgRNA library, and use Fluorescence-Activated Cell Sorting (FACS) to isolate and enrich cells harboring sgRNA constructs. We then perform positive selection screening by exposing the PigGeCKO cell collection to repeat rounds of JEV challenge, retaining the viable (i.e., JEV-resistant) cells from each round. PCR amplification and deep sequencing enable us to detect enrichment among the candidate JEV-infection-associated genes for annotations relating to HSPGs and endoplasmic reticulum-associated protein degradation (ERAD) pathways. We then generate gene knockout (KO) and knockdown cell lines for six of the candidate genes, and successfully confirm their requirement for JEV infection in porcine cells. These newly discovered host genes are potential targets for the development of therapies for the treatment of Japanese encephalitis and porcine diseases caused by JEV, and can also be used in the construction of genetically edited disease-resistant animal models. We anticipate that our benchmark-setting CRISPR/Cas9 screening resources will greatly facilitate basic and applied functional genomics research in pigs.

## Results

### Strategy for identifying genes essential for JEV-induced cell death in pigs

**Fig. 1:**
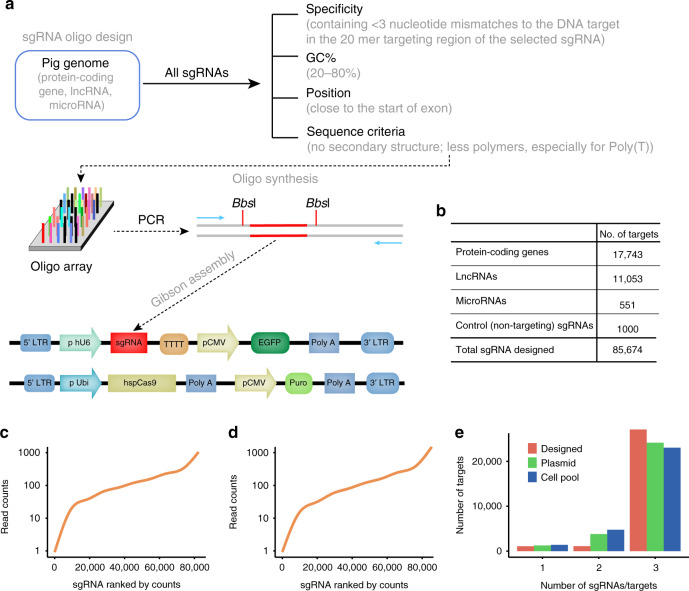
Generation of a porcine genome-wide lentiviral sgRNA library. **a** Pipeline for sgRNA library design and construction. Protein-coding genes from Ensemble, lncRNAs from the domestic-animal lncRNA database (ALDB), and miRNAs from the miRBase database. Primers indicates by blue arrows were used for amplification of the sgRNA targeting sequences from the synthesized oligo array, which were cloned into the lentiviral plasmid. **b** The number of designed sgRNA targets. **c**, **d** Sequencing result of sgRNAs targeting sequences in two CRISPR pooled sgRNA libraries. Plasmid pools (**c**) and sorted mutant cell populations (**d**) containing the whole CRISPR pooled sgRNA library were characterized using next-generation sequencing (NGS). The curve indicates the distribution of sgRNAs. **e** The number of sgRNAs per gene in the genome-wide CRISPR pooled sgRNA library from the designed, plasmid, or mutant cell pools. sgRNA, small guide RNA; PCR, Polymerase chain reaction; hU6, human U6 promoter; Ubi, Ubiquitin promoter; CMV, Human cytomegalovirus promoter; EGFP, Enhanced green fluorescent protein; TTTT, U6 terminator sequence; LTR, long terminal repeat; hspCas9, human codon-optimized *Streptococcus pyogenes* Cas9; Puro, puromycin; LncRNA, Long non-coding RNA; Designed, sgRNA designed by CRISPR-offinder software; Plasmid, the sequencing result of sgRNA library from plasmid pools; Cell pool, the sequencing result of sgRNA library from sorted mutant cell populations. Source data are provided as Supplementary Data files.

The overall development process of the PigGeCKO resources is depicted in the schematic diagram in Fig. 1a, and proceeded as follows. We initially used CRISPR-offinder (v1.2)^26^ to design 85,674 specific and predicted high-efficiency single guide RNAs (sgRNAs), that collectively targeted 17,743 protein-coding genes, 11,053 long ncRNAs (lncRNAs), and 551 microRNAs (miRNAs) in the porcine genome, as well as 1000 negative control sgRNA constructs predicted not to target any porcine genome loci (Fig. 1b, Supplementary Data 1). Three sgRNA constructs were designed for each targeted locus, all loci which met the selection criteria detailed in the Methods were targeted. These designed sgRNA constructs were synthesized as an oligo array, which was employed as the template for PCR amplification of the sgRNA oligos that were subsequently cloned into lentiviral vectors using Gibson assembly.

To test the quality of the PigGeCKO plasmid library, we amplified the cloned sgRNA constructs using PCR, performed deep sequencing, and found that 96.2% (82,426/85,674) of the initially designed and synthesized sgRNA sequences were present in the plasmid library (Fig. 1c, Supplementary Data 2). Although a small fraction of sgRNA were under- or over-represented, ~90% of the sgRNAs were within a range covering a tenfold difference in frequency (Fig. 1c). In parallel with our sgRNA library preparation, we developed a PK-15 cell line that stably expressed high levels of Cas9 (PK-15-Cas9, Clone-#14, Supplementary Fig. 1). We used an sgRNA lentivirus targeting the randomly selected *ANPEP* gene to assess the capacity of this cell line for gene editing (Supplementary Fig. 2a), and found that gene-editing activity tended to be stable ~6–10 days post-infection of the sgRNA-harboring lentivirus in PK-15-Cas9 cells (Supplementary Fig. 2b). We then generated the PigGeCKO lentivirus library by transfecting HEK293T cells with the lentiviral sgRNA plasmids together with helper plasmids. To minimize the chance of inserting multiple sgRNAs into the same PK-15-Cas9 cells, we employed a low multiplicity of infection (MOI) to obtain a transduction rate of around 30% according to a previous study^27^. The lentivirus sgRNA library was subsequently transduced into PK-15-Cas9 cells. We performed FACS-based sorting on the signal from the green fluorescent protein (GFP) reporter, which was included in all PigGeCKO constructs. Then, infected cells were screened for the presence of sgRNA construct sequences by PCR analysis and deep sequencing.

Strikingly, 94.7% (81,095/85,674) of the originally designed sgRNA sequences were retained in the PigGeCKO knockout cell collection (Fig. 1d, Supplementary Data 2). Furthermore, the plasmid library and PigGeCKO cell collection included all three of the designed sgRNA sequences for the majority of the targeted loci in the pig genome (Fig. 1e). Finally, one of the originally designed sgRNA sequences (targeting the *IZUMO3* gene) was randomly selected to evaluate potential off-target effects (Supplementary Fig. 3a). A T7EN I cleavage assay revealed no off-target cleavage for any of the predicted potential off-target sites (Supplementary Fig. 3b). Collectively, this work demonstrates the development of a highly active and specific PigGeCKO resource with high utility for functional genomics research in pigs.

### JEV-induced cell death screening of the PigGeCKO cell collection to identify required host genes

**Fig. 2:**
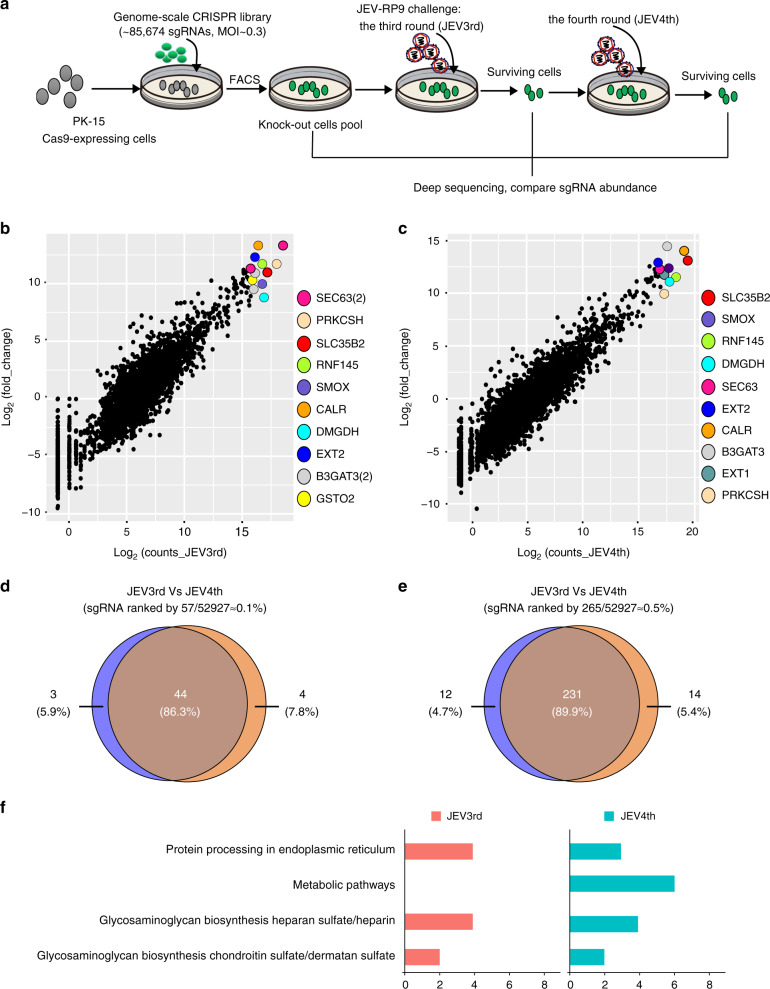
JEV-resistance screen in PK-15 cells using the porcine genome-scale CRISPR/Cas9 knockout cell collection. **a** Workflow and screening strategy for the CRISPR/Cas9 screen. **b**, **c** Scatter plots comparing sgRNA targeting sequences frequencies and extent of enrichment vs. the noninoculated control mutant cell pool for the third or fourth (**c**) rounds of JEV screens after challenge. Counts_JEV3rd and Counts_JEV4th represent the average values of the read counts from paired-end sequencing, respectively. **d**, **e** Venn diagrams showing the overlapping enrichment of specific sgRNAs targeting sequences in the third or fourth rounds of JEV screens after challenge. For **d**, among the top 0.1% of averaged reads for the sgRNAs; for **e**, among the top 0.5%. **f** KEGG pathway enrichment analyses for the top 0.5% ranked sgRNA targets from the third and fourth JEV challenge rounds. JEV, Japanese encephalitis virus; MOI, multiplicity of infection; sgRNA, small guide RNA; JEV3rd, the third challenge round of JEV screening; JEV4th, the fourth challenge round of JEV screening; FACS, Fluorescence-activated cell sorting; JEV-RP9, JEV, genotype 3, strain RP9. Source data are provided as Supplementary Data files.

We then developed a screening strategy, illustrated in Fig. 2a, to identify host genes required for successful JEV infection. To determine the optimal virus level for JEV-induced cell death in PK-15 cells for CRISPR screening, we examined JEV-induced cell death following infection at MOIs of 0, 0.01, 0.05, and 0.1. As the infection dose of JEV was increased, we observed cytopathic effects (CPE) at approximately days 4 post virus infection; phenotypes included the rounding up and enlargement of cells, the formation of syncytia, and the detachment of cells into the medium (Supplementary Fig. 4).

Having established that PK-15 cells are a suitable model for identifying host genes with functions relating to JEV infection, we undertook our screening to identify host genes that modulate susceptibility to JEV-induced cell death. The PigGeCKO cell collection was infected with JEV and incubated for 11 days to enable selection of cells resistant to JEV-induced killing. We conducted four rounds of JEV challenge, employing untreated PK-15-Cas9 cells as a negative control to confirm the cell death caused by JEV infection in each round (Fig. 2a). At an MOI of 0.03, all untreated JEV-infected PK-15-Cas9 cells died, whereas a small number of viable cells from the JEV-infected PigGeCKO cell collection were detected. Surviving cells were collected and used for subsequent JEV challenge rounds, and the sgRNA constructs in surviving cells were PCR amplified and deep sequenced to identify candidate genes.

Focusing on protein-coding genes, our screen found that a total of 2181 unique sgRNA sequences were present in at least ten cells in the third and fourth rounds of JEV challenge. Among the originally designed 52,928 sgRNAs targeting 17,743 protein-coding genes (Supplementary Data 3), after JEV challenge, only 280 of the sgRNA constructs were present in at least 1000 of the total population of analyzed cells (~0.5% of the total analyzed cells) (Supplementary Data 3). The top ten most enriched candidate genes after the third JEV challenge round were (highest-to-lowest) *SEC63*, *PRKCSH*, *SLC35B2*, *RNF145*, *SMOX*, *CALR*, *DMGDH*, *EXT2*, *B3GAT3*, and *GSTO2* (Fig. 2b); the fourth challenge round identified the same enriched genes with the exception of *EXT1* and *GSTO2* (Fig. 2c). When taking into consideration the design of three sgRNAs constructs for each targeted locus of the porcine genome, we found that two sgRNAs for each of the *SEC63* and *B3GAT3* genes were among the most highly enriched sequences after the third rounds of JEV challenge (Fig. 2b, c).

After the third JEV challenge round, there were 57 sgRNA constructs present in at least 10,000 cells, and 219 sgRNA constructs present in at least 1000 cells; after the fourth JEV challenge round, these numbers were 57 and 239 respectively (Supplementary Data 3). Comparison of enriched sgRNAs from the positive selection CRISPR screening revealed that 86.3% of the very highly enriched (i.e., ≥10,000) sequences were common to both the third and fourth challenge rounds, and that 89.9% of the highly enriched (≥1000) sequences were common to both rounds (Fig. 2d, e). These results highlight the capacity for CRISPR-based positive selection screening to consistently identify strong candidate genes. To explore the predicted biological functions of the candidate JEV-resistance genes, we performed KEGG pathway enrichment analyses for top 0.5% ranked sgRNA targets from the third and fourth JEV challenge rounds. These analyses revealed that the candidate JEV-resistance genes were significantly enriched for HSPGs metabolism and for Golgi and endoplasmic reticulum (ER) functions (Fig. 2f). Among these genes, *B3GAT3* and *EXT1* have known roles in JEV replication^28^. We also found a large difference in the abundance of metabolic pathway hits between the third and fourth rounds of viral challenge. We speculate that cell growth may be adversely affected, and cell death may occur more frequently following knockout of genes involved in metabolism, although further study is warranted to verify this possibility. These results indicate that key host factors involved in JEV replication can be identified through multiple rounds of CRISPR screening.

### Knockout of HSPGs pathway-related genes significantly inhibits JEV entry

**Fig. 3:**
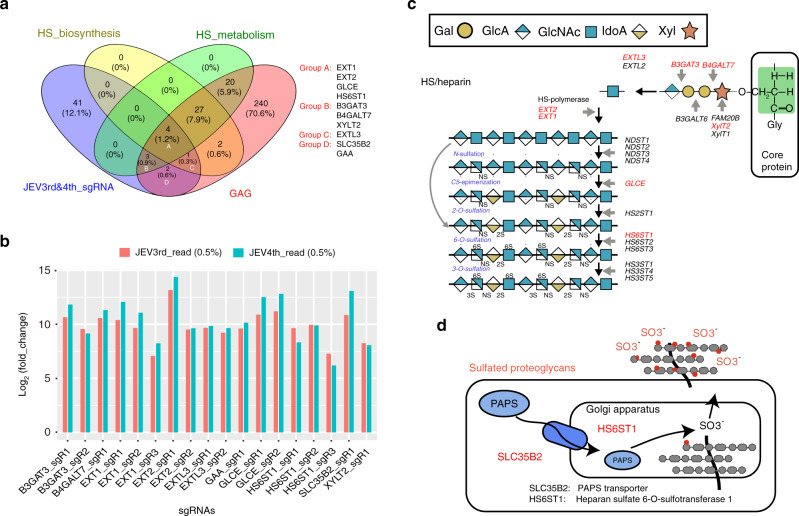
Significant enrichment of specific sgRNAs targeting 10 genes involved in HSPGs synthesis and metabolic pathways. **a** Venn diagram showing the overlapping enrichment of specific sgRNA targeted genes identified in the JEV screen of the cell knockout collection, HSPGs biosynthesis and metabolism, and GAG metabolism pathways. **b** Enrichment of specific sgRNAs targeting 10 genes involved in HSPGs synthesis and metabolic pathways. Only the top 0.5% of sgRNA was counted. **c** Schematic diagram, adapted from Tanaka et al. ^28^, showing the various classes of chemical components comprising HSPGs with known enzymes for their biosynthesis. Red indicates targeted genes identified in this study. **d** Schematic diagram, adapted from Blondel et al. ^34^, showing the known components and localization information for the sulfurylation modifications known to occur for some HSPGs. Red indicates targeted genes identified in this study. JEV, Japanese encephalitis virus; HS, Heparan sulfate; GAG, Glycosaminoglycan; HSPGs, heparan sulfate proteoglycans; PAPS, 3’-Phosphoadenosine-5’-phosphosulfate; JEV3rd, the third challenge round of JEV screening; JEV4th, the fourth challenge round of JEV screening. Source data are provided as a Supplementary Data file.

There are conflicting findings from previous studies of possible functional roles for heparan sulfate pathway proteins as cellular attachment factors during initiation of JEV infection^29,30^. HSPGs encompass a diverse class of proteins defined by the substitution with heparan sulfate glycosaminoglycan (GAG) polysaccharide chains^31,32^. Our genome-scale CRISPR screen for JEV-infection related genes indicated that 10 genes associated with HSPGs metabolism were among the most highly enriched sgRNA targeted genes: *EXT1*, *EXT2*, *GLCE*, *HS6ST1*, *B3GAT3*, *B4GALT7*, *XYLT7*, *EXTL3*, *SLC35B2*, and *GAA* (Fig. 3a). Among these genes, *SLC35B2*, *EXT1*, and *EXT2* were ranked in top 10 from both the third and fourth JEV challenge rounds. Notably, the *EXT1* and *HS6ST1* genes were each targeted by three separate sgRNA constructs, all of which were highly enriched, clearly indicating potential JEV-infection-related functions (Fig. 3b). HSPGs synthesis and sulfation is driven by >20 different genes^28^; as shown in Fig. 3c, the significant enrichment of specific sgRNAs identified seven genes potentially involved in HSPGs synthesis and metabolic pathways, and two genes potentially involved in sulfurylation modifications of HSPGs^33,34^ in porcine cells (Fig. 3d).

**Fig. 4:**
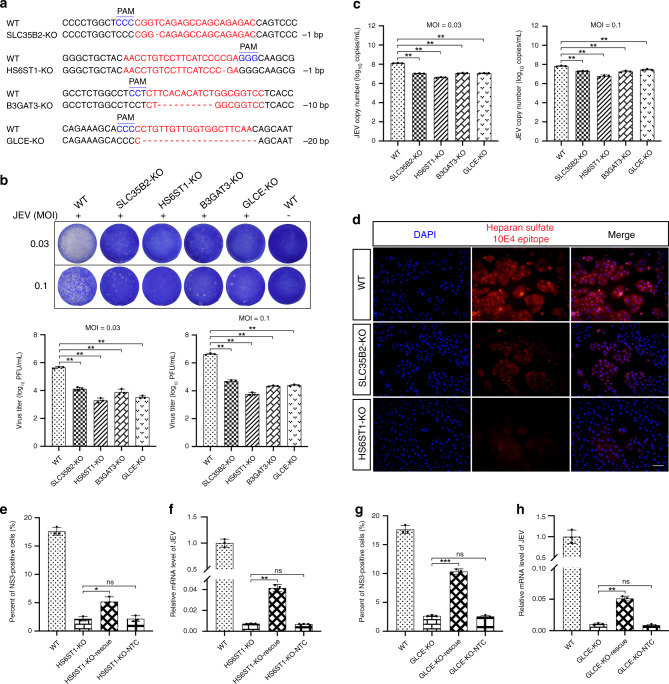
Knockout of genes coding for the HSPGs pathway proteins SLC35B2, HS6ST1, B3GAT3, and GLCE significantly inhibits JEV replication in PK-15 cells. **a** Alignment of the nucleic acid sequences of clonal KO cells of *SLC35B2*, *HS6ST1*, *B3GAT3*, and *GLCE* with WT cells. sgRNA targeting sites are highlighted in red. The red characters “-” indicate the deleted bases in the KO cells. PAM sites are indicated in blue letters. **b** Virus plaque assays for determination of viral concentration in clonal *SLC35B2*, *HS6ST1*, *B3GAT3*, and *GLCE* KO cell lines following infection with JEV at an MOI of 0.03 or 0.1. **c** Absolute quantitative real-time PCR for determination of JEV copy number in clonal *SLC35B2*, *HS6ST1*, *B3GAT3*, and *GLCE* KO cell lines following infection with JEV at an MOI of 0.03 or 0.1. **d** Detection of HSPGs sulfurylation level in clonal *SLC35B2* and *HS6ST1* KO cell lines by immunofluorescence. Scale bar, 200 μm. **e**, **f** Rescue assays for ectopic expression of HS6ST1 in HS6ST1-deficient cells. HS6ST1-KO-rescue: Transfection of pcDNA3.1-HS6ST1 vector in HS6ST1-deficient cells; HS6ST1-KO-NTC: Transfection of pcDNA3.1 empty vector in HS6ST1-deficient cells. **g**, **h** Rescue assays for ectopic expression of GLCE in GLCE-deficient cells. GLCE-KO-rescue: Transfection of pcDNA3.1-GLCE vector in GLCE-deficient cells; GLCE-KO-NTC: Transfection of pcDNA3.1 empty vector in GLCE-deficient cells. Detection of NS3 by immunofluorescence microscopy, the original data came from Supplementary Fig. 10a (**e**) and Supplementary Fig. 10c (**g**), respectively. RT-qPCR assay (**f**, **h**) for determination of relative mRNA level of JEV *C* gene in rescue assays of HS6ST1 and GLCE, respectively. PAM, protospacer adjacent motif; JEV, Japanese encephalitis virus; MOI, multiplicities of infection; hpi, hours post-infection; KO, knockout; WT, wild-type; DAPI, 4’, 6-diamidino-2-phenylindole. Data are represented as means ± S.D.; *n* = 3 (**b**, **c**, **e**, **f**, **g**, **h**). *P < 0.05; **P < 0.01; ***P < 0.001; ns, no significant. *P* values were determined by two-sided Student’s *t*-test. Source data are provided as a Source Data file.

Building from these initial candidate hits, we first generated KO cells of *SLC35B2*, *HS6ST1*, *B3GAT3*, and *GLCE* using the CRISPR/Cas9 editing system. Before validating the specific hits, initial evaluations showed that the integrated expression of a randomly selected scrambled sequence negative control sgRNA did not affect the normal replication of JEV (Supplementary Fig. 5). Subsequently, we found that individual knockouts of *SLC35B2*, *HS6ST1*, *B3GAT3*, and *GLCE* exhibited inhibition of cell death induced by JEV infection at an MOI of 0.03, although the suppression of cell death was not complete at high-dose JEV challenges (MOI = 1) (Supplementary Fig. 6a and b). Sanger sequencing confirmed that each of these KO cell lines had one or more nucleotide deletions predicted to cause a frameshift mutation in the coding regions of the targeted gene (a non-integer multiple of 3) (Fig. 4a, Supplementary Fig. 7a). Moreover, the results of EdU fluorescence assays showed no difference in the rates of cell proliferation between corresponding KO and wild-type (WT) PK-15 cells (Supplementary Fig. 7b).

Then, viral loads in JEV-infected *SLC35B2*, *HS6ST1*, *B3GAT3*, and *GLCE* KO cells and in WT PK-15 cells were measured at 18 hpi (hours post-infection) by plaque assay, which only using JEV at an MOI of 0.03 or 0.1 (Fig. 4b). In agreement with the reduced viral loads observed in KO cells, the results from the immunofluorescence assays showed that the expression of the JEV-encoded NS3 protein in all four KO cell lines was modestly reduced or undetectable following JEV infection at both MOIs of 0.03 and 0.1 (Supplementary Fig. 8). Next, cell cultures were sampled at 18 hpi for quantification of JEV genome copy number based on absolute quantitative PCR analysis targeting the *C* gene of JEV. These analyses revealed that knockout of these four HSPGs-related genes significantly inhibited JEV replication (12 hpi) (Fig. 4c). Importantly, use of an antibody against Heparin/Heparan Sulfate antibody (10E4) to conduct immunofluorescence assays revealed that knockout of the *SLC35B2* and *HS6ST1* genes resulted in a significant reduction in the HSPGs sulfurylation level compared to WT cells (Fig. 4d). This observation clearly suggests that sulfurylation modifications of HSPGs can significantly and functionally impact the interaction between JEV and HSPGs in PK-15 cells, potentially during viral entry.

To exclude the possibility of other defects that may result in the inhibition of JEV infection in these KO cells, we performed rescue experiments to demonstrate complementation by the intact genes. Surprisingly, we found that knockout of *SLC35B3* or *B3GAT3* resulted in complete inhibition plasmid delivery using lipofection transfection reagent, thus preventing ectopic expression of *SLC35B3* and *B3GAT3* in corresponding KO cells (Supplementary Fig. 9). Thus, the other two genes, *HS6ST1* and *GLCE* were selected for further study. We found that the ectopic expression of HS6ST1 or GLCE in corresponding KO cells by rescue assays resulted in partial recovery of JEV replication (Fig. 4e–h, Supplementary Fig. 10). As shown in Fig. 4e, the proportion of NS3-positive cells was significantly increased during restoration of HS6ST1 expression in HS6ST1-deficient cells. And the proportion of NS3-positive cells was also significantly increased during restoration of GLCE expression in GLCE-deficient cells (Fig. 4g). Further analysis revealed that the knockout of *HS6ST1* or *GLCE* can fully support viral replication by infectious JEV cDNA clone system studies (Supplementary Fig. 11). Subsequent RNAi assays showed that both single and double knockdown of HSPGs pathway genes coding SLC35B2, HS6ST1, B3GAT3, and GLCE proteins led to significant inhibition of JEV replication in PK-15 or ST cells (Supplementary Figs. 12–14). These results were consistent with CRISPR-mediated knockout of HSPGs pathway candidate genes, thus further confirming that JEV replication requires a complete HSPGs pathway. Collectively, these results indicate that HSPGs can only act as a cellular adhesion factor or cofactor that mediates JEV entry.

### EMC3 is required for JEV replication

**Fig. 5:**
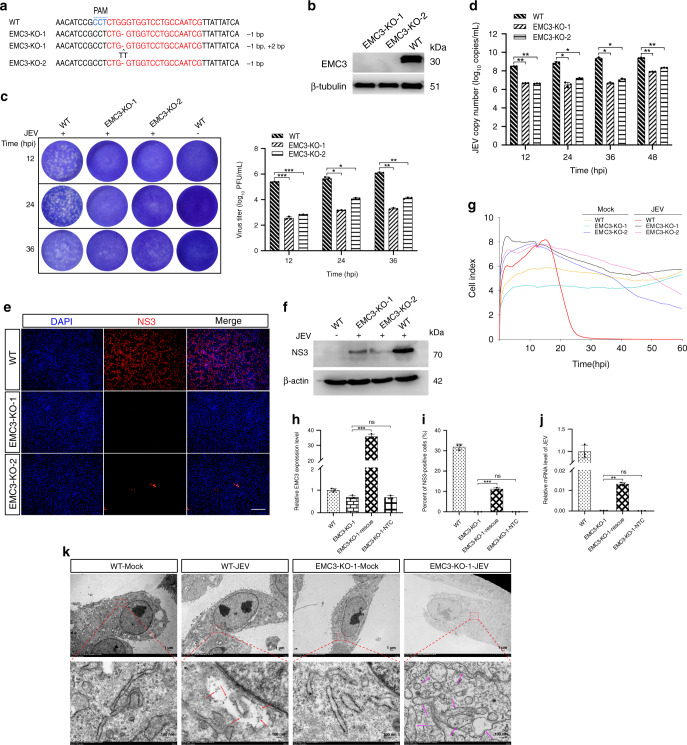
The EMC3 is required for JEV replication. **a** Alignment of the nucleic acid sequences of two clonal KO cells of *EMC3* with WT cells. sgRNA targeting sites are highlighted in red. The red characters “-” indicate the deleted bases, and black “^” characters indicate the inserted bases in KO cells. PAM site is indicated in blue letters. **b** Western blot analysis of EMC3. **c** Virus plaque assays. **d** Absolute quantitative real-time PCR. **e** Immunofluorescence for detection of NS3 expressed in *EMC3* KO cell lines following infection with JEV. Scale bar, 200 μm. **f** Western blot was performed to examine the expression of the NS3 in *EMC3* KO cell lines following infection with JEV. **g** Real-Time Cell Analyzer assay was performed to record the cell survival curves. **h**, **i**, **j** Rescue assays for ectopic expression of EMC3 in KO cells. RT-qPCR assay for determination of relative mRNA level of *EMC3* (**h**); Detection of NS3 by Immunofluorescence, the original data came from Supplementary Fig. 16a (**i**). RT-qPCR assay for determination of relative mRNA level of JEV *C* gene (**j**). EMC3-KO-1-rescue: Transfection of pcDNA3.1-EMC3 vector in KO cells; EMC3-KO-1-NTC: Transfection of pcDNA3.1 empty vector in KO cells. **k** Evaluation of the effects of *EMC3* knockout on virus particle assembly by negative-staining electron microscopy. Numerous scattered virus-like particles were present in the diluted cisternae of the ER (Red arrow). The pink arrow indicates KO cells displayed dramatic changes in ER morphology after JEV infection. Scale bar, 1 µm or 100 nm. PAM, protospacer adjacent motif; JEV, Japanese encephalitis virus; Mock, non-infected cells was included as negative control; MOI, multiplicities of infection; hpi, hours post-infection; KO, knockout; WT, wild-type; DAPI, 4’, 6-diamidino-2-phenylindole; kDa, kilodalton. The experiments were repeated three times with similar results and representative results shown (**k**). Data are represented as means ± S.D.; *n* = 3 (**c**, **d**, **h**, **i**, **j**). *P < 0.05; **P < 0.01; ***P < 0.001; ns, no significant. *P* values were determined by two-sided Student’s *t*-test.

The endoplasmic reticulum membrane complex (EMC) is known to be required for infection by flaviviruses, which have RNA genomes^35,36^. However, it is unclear whether EMC family genes are involved in JEV replication. Interestingly, our genome-scale CRISPR JEV-infection screen showed that *EMC3* and *EMC6* genes, both of which encode ER membrane protein complex subunits, were ranked 44 and 34 among the candidate hits in the fourth JEV challenge round, respectively (Supplementary Data 3). A previous study identified that knockout of the *EMC6* led to significant inhibition of JEV replication in human HEK293T cells^37^, which was consistent with our findings in pig. As such, we only generated two independent *EMC3* null cell lines using CRISPR/Cas9. Sanger sequencing confirmed the presence of 1 or 2 bp deletions or insertions (a non-integer multiple of 3) in both *EMC3* KO cell lines (Fig. 5a, Supplementary Fig. 15a), and immunoblotting confirmed that the EMC3 protein was not expressed in cells of either KO line (Fig. 5b). Moreover, the results of EdU fluorescence assays showed no difference in the rates of cell proliferation between *EMC3* KO and WT PK-15 cells (Supplementary Fig. 15b).

Subsequently, viral concentrations in JEV-infected *EMC3* null and WT PK-15 cells were determined at 12, 24, and 36 hpi by both plaque assay and quantification of JEV genome copy number based on absolute quantitative PCR analysis using a pair of primers targeting the *C* gene of JEV. Together, these analyses revealed that knockout of the *EMC3* gene significantly inhibited JEV replication (12 hpi) at an MOI of 1 (Fig. 5c, d). Both JEV-infected *EMC3* KO cell lines possessed substantially reduced levels of viral NS3 protein expression as determined via immunofluorescence analysis (Fig. 5e) and immunoblotting (Fig. 5f). Next, the ability of EMC3-deficient cells to resist JEV-induced death at an MOI of 1 was evaluated. The result from cultures grown using a Real-Time Cell Analyzer assay verified that EMC3-deficient cells were able to completely resist JEV-induced PK-15 cell death (Fig. 5g). Subsequently, we found that rescue assays for ectopic expression of EMC3 in EMC3*-*deficient cells resulted in partial recovery of JEV replication (Fig. 5h–j, Supplementary Fig. 16a). As shown in Fig. 5j, the proportion of NS3-positive cells was significantly increased during restoration of EMC3 expression in EMC3-deficient cells. Further analysis revealed that knockdown of *EMC3* significantly inhibited JEV replication in both PK-15 and ST cells (Supplementary Fig. 17, 18). In a final step, we further investigated the effects of *EMC3* knockout on virus particle assembly in EMC3-deficient cells by negative-staining electron microscopy. In WT cells infected with JEV, virus particles were observed in the ER, but were not observed in the ER of EMC3-deficient cells after JEV infection (Fig. 5k). Compared with the WT cells, at 24 h (hrs) post-JEV infection, EMC3-deficient cells displayed dramatic changes in membrane morphology, potentially related to the observed suppression of JEV transcription and/or translation (Fig. 5k, Supplementary Fig. 19). Collectively, these results demonstrate that the *EMC3* is required for JEV-induced PK-15 cell death.

### CALR is required for JEV replication

**Fig. 6:**
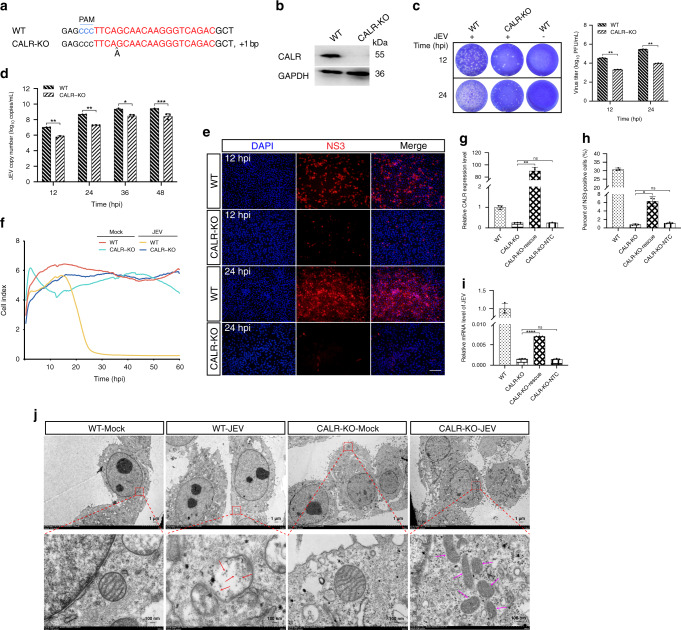
The CALR protein of the endoplasmic reticulum lumen is required for JEV replication. **a** Alignment of the nucleic acid sequences of KO cells of *CALR* with WT cells. sgRNA targeting sites are highlighted in red. The black “^” characters indicate the inserted bases in KO cells. PAM site is indicated in blue letters. **b** Western blot analysis of CALR in KO and WT cells. **c** Virus plaque assays. **d** Absolute quantitative real-time PCR. **e** Immunofluorescence for detection of NS3 expressed in *CALR* KO cell line following infection with JEV. Scale bar, 200 μm. **f** Real-Time Cell Analyzer assay was performed to record the cell survival curves. **g**, **h**, **i** Rescue assays for ectopic expression of CALR in KO cells. RT-qPCR assay for determination of relative mRNA level of *CALR* (**g**); Detection of NS3 by Immunofluorescence, the original data came from Supplementary Fig. 16b (**h**). RT-qPCR assay for determination of relative mRNA level of JEV *C* gene for restoration of CALR expression in KO cells following infection with JEV (**i**). CALR-KO-rescue: Transfection of pcDNA3.1-CALR vector in KO cells; CALR-KO-NTC: Transfection of pcDNA3.1 empty vector in KO cells. **j** Evaluation of the effects of *CALR* knockout on virus particle assembly by negative-staining electron microscopy. Numerous scattered virus-like particles were present in the mitochondrial (Red arrow). The pink arrow indicates KO cells displaying dramatic changes in mitochondrial morphology after JEV infection. Scale bar, 1 µm or 100 nm. PAM, protospacer adjacent motif; JEV, Japanese encephalitis virus; Mock, non-infected cells was included as negative control; MOI, multiplicities of infection; hpi, hours post-infection; DAPI, 4’, 6-diamidino-2-phenylindole; KO, knockout; WT, wild-type; kDa, kilodalton. The experiments were repeated three times with similar results and representative results shown (**j**). Data are represented as means ± S.D.; *n* = 3 (**c**, **d**, **g**, **h**, **i**). *P < 0.05; **P < 0.01; ***P < 0.001; ****P < 0.0001; ns, no significant. *P* values were determined by two-sided Student’s *t*-test. Source data are provided as a Source Data file.

Among the top 10 ranked genes in the genome-scale CRISPR screen for JEV-infection screening candidates was a gene known to function in intracellular calcium homeostasis: *CALR*. To explore the potential function of *CALR* in mediating JEV replication, *CALR* KO cells was generated by CRISPR/Cas9 technology. Sanger sequencing showed that the selected CLAR-deficient cells have a 1 bp insertion (Fig. 6a, Supplementary Fig. 15a), and immunoblotting confirmed that the CALR protein was not expressed in the KO cells (Fig. 6b). Moreover, the results of EdU fluorescence assays showed no difference in the rates of cell proliferation between *CALR* KO and WT PK-15 cells (Supplementary Fig. 15b).

Next, plaque assays and absolute quantitative real-time PCR were used to measure viral concentrations in JEV-infected *CALR* null and WT PK-15 cells at 12 and 24 hpi. Concurrently, JEV-infected cell cultures were harvested at 12, 24, 36, and 48 hpi, and viral RNAs were extracted from cell suspensions and cDNAs were synthesized as absolute quantitative PCR template. As shown in Fig. 6c, d, knockout of the *CALR* gene significantly inhibited JEV replication at an MOI of 1. Moreover, immunofluorescence results showed that the NS3 protein was only weakly expressed at 12 and 24 hpi in JEV-infected *CALR* null cell lines (Fig. 6e). Next, the ability of CALR-deficient cells to resist JEV-induced death at an MOI of 1 was evaluated. Results of the cell proliferation assay and Real-Time Cell Analyzer assay showed that, compared to WT cells, knockout of *CALR* can also confer resistance to JEV-induced death (Fig. 6f). Subsequently, we found that rescue assays for ectopic expression of CALR in CALR*-*deficient cells resulted in partial recovery of JEV replication (Fig. 6g–i, Supplementary Fig. 16b). As shown in Fig. 6h, the proportion of NS3-positive cells was significantly increased during restoration of CALR expression in CALR-deficient cells. Further analysis revealed that knockdown of *CALR* significantly inhibited JEV replication in PK-15 or ST cells (Supplementary Figs. 17, 18). We also further investigated the effects of *CALR* knockout on virus particle assembly in *CALR* null cells by negative-staining electron microscopy. In WT PK-15 cells infected with JEV, virus particles were observed in the mitochondria, but not in the mitochondria of CALR-deficient cells after JEV infection (Fig. 6j). Furthermore, these results showed that JEV infection leads to diminished mitochondrial crista in WT cells, but which are preserved by CALR-deficient cells (Fig. 6j, Supplementary Fig. 20). These results indicate that CALR is required for JEV replication.

## Discussion

**Fig. 7:**
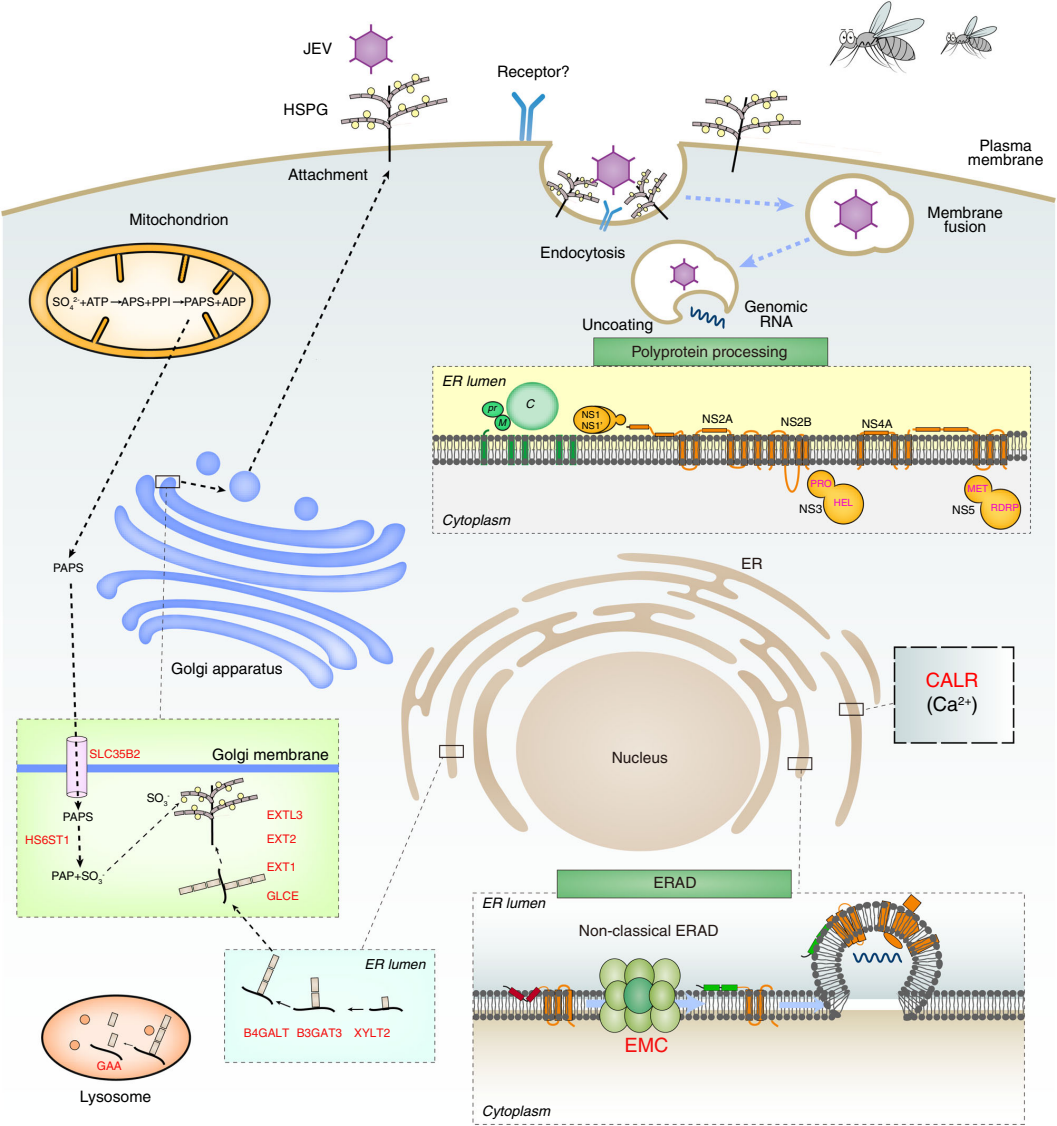
A proposed model for JEV entry and replication in porcine cells. The JEV-infection cycle starts with binding to co-factors HSPGs, and/or unknown cellular receptors, followed by viral entry to enable replication. The EMC complex protein (EMC3 and EMC6) and CALR calcium-binding protein of the ER lumen are involved in JEV replication of host cells. Subsequently, the JEV RNA genome is replicated, viral particles are matured and packaged, and are released from cells. JEV, Japanese encephalitis virus; HSPG, heparan sulfate proteoglycan; PAPS, 3′-Phosphoadenosine-5′-phosphosulfate; PAP, 3′-phosphoadenosine-5′-phosphate; ATP, Adenosine triphosphate; ADP, Adenosine diphosphate; APS, Adenosine 5′ phosphosulfate; PPI, pyrophosphate; ER, endoplasmic reticulum; ERAD, endoplasmic reticulum-associated protein degradation; EMC, endoplasmic reticulum membrane protein complex.

Our results highlight the power of CRISPR/Cas9-based screening for functional analyses in pigs, and we present in Fig. 7 a preliminary proposed model for JEV entry and replication in porcine cells based on our findings. The CRISPR screens recovered JEV entry cofactor HSPGs-related host factors (SLC35B2, HS6ST1, EXT1, EXT2, GLCE, B3GAT3, B4GALT7, XYLT7, and EXTL3), as well as multiple host factors involved in calcium homeostasis (CALR), and transmembrane protein processing and maturation (EMC3 and EMC6). In addition, a large number of other candidate host factors involved in JEV infection of host cells were identified with our CRISPR screen, warranting further investigation. Furthermore, to determine which genes were essential for cell growth and survival, we felt the best strategy to achieve this was to apply negative selection to CRISPR screening. In this study, we aimed to determine host factors that participate in JEV replication, which is a positive selection. Therefore, it was difficult to determine which of these genes are essential for cell growth. The functions of candidate genes were finally tested through knockout or knockdown assays, confirming that these genes identified by CRISPR screening were reliable.

While genome-scale CRISPR/Cas9 mutagenesis methods obviously facilitate gene functional studies in both cellular and animal models^19–24^, and the ability to programmably target the entire coding or regulatory genome represents a significant advance over spontaneous or random mutagenesis. Genome-scale CRISPR/Cas9 approaches share with classical forward genetic screens the requirement for an assay to enrich for cells exhibiting the phenotype of interest^38–40^. JEV causes encephalitis in humans and reproductive disorders in pigs, the latter leading to substantial economic losses^10^. In the present study, we developed a series of porcine genome-scale CRISPR/Cas9 knockout library resources that facilitate the pooled screening of genes that prevent JEV invasion or replication, thereby inhibiting JEV-induced cell death in porcine cells. Furthermore, we demonstrated how lentiviral delivery of a PigGeCKO library targeting 17,743 protein-coding genes enables positive selection screening for JEV-replication-associated host genes.

The first candidate genes examined from this screen are known to function in HSPGs metabolism, specifically in the synthesis and modification of heparan sulfate chains in normal cells. The murine homolog of EXT1 protein is known to be localized to the Golgi apparatus^41^, where it binds with EXT2 to form a complex known to modify heparan sulfate^41^. EXT1, EXT2, and EXTL3 together contribute to heparan sulfate chain elongation^42^. In mice, GLCE is an epimerase enzyme required for the biosynthesis of HSPGs, which are composed of a core protein and one or more heparan sulfate glycosaminoglycan chains^43^. The B3GAT3 enzyme catalyzes the formation of glycosaminoglycan-protein linkages by glucuronic acid transfer, which is the final step in the biosynthesis of proteoglycan-linked regions^28^. A previous study identified that knocking out the *B3GAT3* gene can significantly inhibit JEV replication^28^, a conclusion that was further confirmed by our genome-scale CRISPR screening and subsequent hypothesis-driven functional analyses. A different study showed that the reduced activity of the B4GALT7 enzyme is associated with a reduced substitution of the proteoglycans decorin and biglycan (both of which have glycosaminoglycan carbohydrate chains) and alterations in heparan sulfate biosynthesis^44^.

Our work strongly supports that HSPGs pathway genes can mediate JEV replication in porcine cells. Previous studies have shown that the *SLC35B2* gene product is located in the microsomal membrane and functions to transport 3’-Phosphoadenosine-5’-phosphosulfate (PAPS) from the cytosol (where it is synthesized) into the Golgi lumen^44^, whereas the HS6ST1 sulfotransferase enzyme uses PAPS as a substrate for heparan sulfate biosynthesis^45,46^. Accordingly, our immunofluorescence assays showed that *SLC35B2* or *HS6ST1* gene knockout in PK-15 cells significantly reduced the extent of sulfurylation modification to heparan sulfate. Therefore, we believe that sulfurylated HSPG serves as an adjunct or adhesion factor during JEV invasion of host cells. It should be noted that our initial screening efforts were based on relatively low MOI JEV challenges of PK-15 mutant cells. In CRISPR screening assays, we selected a relatively low JEV-infection dose in order to screen for as many candidate host factors for participation in JEV replication as possible. In some cases, knockout of genes from the HSPGs pathway only led to inhibition of relatively low dose JEV infections, but did not prevent replication of relatively high doses of JEV (Supplementary Fig. 6a and b). A previous study showed that some spatially adjacent residues within the JEV domain III are involved in heparin binding^47^. These results indicate that JEV and HSPG may interact with each other on the surface of host cells. In addition, it has already been known that highly sulfated forms of heparin sulfate can bind to the envelope protein and are involved in initial flavivirus attachment^32^. Therefore, in combination with the results of our Real Time Cell Analysis (RTCA) assays (Supplementary Fig. 6a and b), we speculate that HSPGs may only serve as an adjunct or attachment factor for JEV entry. Thus, knocking out HSPGs pathway genes can only effectively inhibit cell death induced by low dose of JEV infection. However, for high-dose JEV infection, it is possible that JEV can directly enter host cells through other unknown pathways. Although these experiments succeeded in identifying many candidate host factors involved in JEV replication at a relative low dose, future CRISPR screening efforts employing a higher dose viral challenge would further confirm the key host factors that participate in JEV high-dose infections.

The second host cellular process implicated in JEV infection involves the EMC3 subunit of the ER membrane protein complex, which is involved in ER-mitochondrial membrane tethering and required to facilitate lipid transfer from the ER to the mitochondrial membrane, impacting nearly all aspects of cell physiology^48^. Previous research has found that the ER membrane protein complex is a transmembrane domain insertase, thus, loss of EMC complex proteins cause ER stress and altered protein trafficking^49^. For ZIKV, this complex was required for viral protein accumulation in a cell line harboring a ZIKV replicon^50^. Our CRISPR screen showed that knockout of *EMC3* and *EMC6* can inhibit JEV-induced cell death. In particular, knockout or knockdown of the *EMC3* gene significantly inhibited the replication of JEV in PK-15 or ST cells. EMC3 and EMC6 are associated with the ERAD pathway, which may participate in the secretory protein quality control processes that guide the removal of aberrantly folded proteins from the ER. Thus, whether the EMC subunits participate in JEV protein biogenesis, misfolding, or direct interaction with JEV particles or JEV-encoded proteins requires further study. For example, by using small molecule inhibitors of ER stress.

The final gene we examined via follow-up hypothesis-driven studies was *CALR*, which encodes a multifunctional soluble protein that can bind Ca^2+^ ions. Knockout of *CALR* resulted in a strong JEV-resistance phenotype. Proper folding in the ER is a prerequisite for the correct localization and function of most secreted transmembrane proteins^51^, In addition, previous studies have found that *CALR* and *calnexin* (CANX) chaperones mediate nascent glycoprotein folding in the ER^52,53^. Thus, we hypothesize that *CALR* appears to be an essential gene that links JEV replication to downstream cell death pathways, possibly due to calcium homeostasis disequilibrium. For these reasons, CALR null cells represent a highly useful model for studying the relationship between calcium ion homeostasis and JEV infection. Additional studies in this area are currently ongoing.

While this study validated six genes selected with follow-up knockout studies and infection assays, our positive selection screening strategy yielded many candidates that may function in JEV infection and as such merit further investigation. We plan to extend the screening described herein to achieve full genome saturation to increase the search scope for host factor genes to further deepen our understanding of the multifaceted and spatiotemporally programmed interactions between JEV and host cells. To further verify that the required host factors we identified as necessary for JEV infection of PK-15 cells contribute the same roles in other cells, we also selected the JEV-susceptible ST cell line for RNAi-based knockdown assays. These experiments confirmed that individual knockdown of all six genes also led to significant inhibition of JEV replication in ST cells, thus suggesting that the functions of these genes are conserved across different types of porcine cells.

It is important to consider that some genes may be necessary for normal cell growth, so their knockout may inhibit cell proliferation, which would prevent detection of required genes. JEV is an RNA virus and belongs to the flaviviridae family, and previous studies in humans using CRISPR-based screening strategies have identified many required host genes for cell death induced by DENV, ZIKV, WNV, YFV, and HCV; it is notable that few of these genes appear to overlap^20–24^. On one hand, the identified genes may differ owing to heterogeneities in the various cell types or species; on the other hand, perhaps the host factors for JEV infection simply differ greatly from other flaviviridae family viruses. Our results highlight the complexity of JEV entry, replication, packaging, and release from host cells, yet also lead the way for a variety of hypothesis-driven basic biological and medical studies to deepen our understanding of this complex process.

## Methods

### Plasmids construction

The lenti-Cas9-Puro and lenti-sgRNA-EGFP vectors were kindly provided by Professor Xingxu Huang at ShanghaiTech University. The lenti-Cas9-Puro vector was used for generation of the Cas9-expression cell line (PK-15-Cas9). To construct the lentiviral sgRNA vector, paired oligonucleotides of sgRNA (50 μM per oligo) were annealed and cloned into lenti-sgRNA-EGFP which was linearized with *Bbs*I (Supplementary Data 4). To perform rescue experiments, the encoding region of *SLC35B2*, *HS6ST1*, *B3GAT3*, *GLCE*, *EMC3*, and *CALR* were PCR amplified using cDNA from PK-15 cells as a template, with forward primer flanked by *Nhe*I restriction sites and reverse primer flanked by *Xba*I restriction sites of pcDNA3.1(+) (Thermo Fisher Scientific), respectively. Then, PCR products were digested and cloned into the *Nhe*I and *Xba*I sites to generate pcDNA3.1-SLC35B2, pcDNA3.1-HS6ST1, pcDNA3.1-B3GAT3, pcDNA3.1-GLCE, pcDNA3.1-EMC3, and pcDNA3.1-CALR vectors, respectively. All plasmids were confirmed by Sanger sequencing (Tsingke). All primer sequences are listed in Supplementary Data 4.

### Genome-wide porcine sgRNA library design

Three sgRNAs were designed against each protein-coding gene, lncRNA, and miRNA using software of CRISPR-offinder (version 1.2, http://www.biootools.com^26^). Sequences of protein-coding genes, lncRNA, and miRNA, were found from databases of Ensemble (version 10.2, www.ensembl.org/index.html), ALDB (http://202.200.112.245/aldb^54^), and miRBase (www.mirbase.org^55^), respectively. Briefly, the selected sgRNAs were weighted based on targeting the first 50% of the open reading frames and minimizing potential off-target sites. The maximum number of mismatches allowed up to three nucleotides to the DNA target in the 20 mer targeting region of selected sgRNAs, targeting the miRNA hairpin region, and to avoid overlap between sgRNAs in the same given targets.

### Construction of a genome-wide sgRNA library plasmid

The sgRNA library was synthesized using CustomArray 90 K arrays (CustomArray Inc.), and amplified by PCR using Phusion High-Fidelity PCR Master Mix with HF Buffer (NEB) to produce sub-pools for Gibson assembly (NEB). The PCR reaction was performed in a Veriti™ 96-Well Thermal Cycler (Thermo Fisher Scientific) with 16 cycles. In total, 40 PCR reactions were performed using 50 ng of oligo pool per 50 μL of reaction volume. The PCR products were mixed and purified using a MinElute PCR purification Kit (QIAGEN), and then ligated into the linearized lenti-sgRNA-EGFP vector using Gibson assembly. The ligation mixtures were transformed into Trans1-T1 Phage Resistant Chemically Competent Cells (Transgen). To achieve sufficient coverage, parallel transformations were performed, counting the number of colonies to reach 200-times total sgRNAs in the library. The sgRNA library plasmids were extracted with a Plasmid Plus Maxi Kit (QIAGEN). The library plasmids were amplified using PrimeSTAR GXL DNA Polymerase (Takara) with 16 reaction cycles. PCR products were purified using a QIAquick Gel Extraction Kit (QIAGEN) and then analyzed by high-throughput sequencing to examine the sgRNA coverage in the library plasmids. All primers for constructing sgRNA expression vector are listed in Supplementary Data 4.

### Cell culture and transfection

PK-15, HEK293T, and BHK-21 cell lines were purchased from the Cell Bank of the Chinese Academy of Sciences (Shanghai, China), and ST (CRL-1746^™^) cell line was purchased from ATCC®(USA). The cell lines were then subjected to mycoplasma detection. For all experiments, cells were maintained in Dulbecco’s Modified Eagle Media (DMEM) supplementing with 10% fetal bovine serum (FBS), 100 U/mL penicillin, 100 μg/mL streptomycin and incubated at 37 °C with 5% CO_2_. For transfection assays, PK-15 cells were seeded into 6-well plates and transfected (approximately 80% confluent) with 2 μg plasmid DNA using JetPRIME (PolyPlus) according to the manufacturer’s instructions. At 48-h post-transfection, cells were incubated with JEV-PR9 strain at 37 °C with 5% CO_2_, which was isolated from *Culex tritaeniorhynchus*. At 2-h after incubation, inoculum was removed, and replaced with 2 mL of fresh DMEM containing 10% FBS and 1% penicillin–streptomycin. After 18 h of incubation, immunofluorescence assay and qRT-PCR assay were conducted.

For transfections of infectious JEV cDNA clone, the JEV97 and pCAGGS vectors were kindly provided by Professor Shengbo Cao at Huazhong Agricultural University. The full-length cDNA copy of the JEV genome was cloned into JEV97 vector under the control of T7 promoter. T7 RNA polymerase was expressed by pCAGGS vector. One day before transfection, candidate genes KO and WT PK-15 cells were seeded into 6-well plates, and transfected (~80% confluent) with 1 μg of JEV97 plasmid and 1 μg pCAGGS plasmid using JetPRIME according to the manufacturer’s instructions. At 6-h after transfection, media was removed, and replaced with grown media containing 2% FBS and 1% penicillin–streptomycin. At the third day after incubation, immunofluorescence assay was conducted.

### Generation of Cas9-expression cell line

PK-15 cells were transduced with Cas9-puro lentivirus. At the third day after transduction, cells were changed to fresh growth medium containing 3 μg/mL puromycin. Antibiotics resistant cells were collected and reseeded into 100 mm dishes at a concentration of 100 cells per dish to generate single-cell clones. At the seventh day after antibiotics selection, the single-cell clones were tested for Cas9 expression by immunoblotting. In addition, the *ANPEP* gene targeted by a sgRNA was used to screen cell lines with high expression level and activity of Cas9. The resulting cell lines were designated as PK-15-Cas9.

### sgRNA library lentivirus production and transduction

To produce lentivirus, co-transfection of 12 μg of the library plasmid, 4 μg of pMD2.G plasmid (Addgene), and 8 μg of psPAX2 (Addgene) plasmid per 100 mm dish by using JetPRIME (PolyPlus) according to the manufacturer’s instructions. At 60 hrs post-transfection, the cell supernatants were collected, filtered by using a 0.45 μm low protein binding membrane (Millipore), and then centrifuged at 153,700 × *g* and 4 °C for 2.5 h. The virus pellets were resuspended in phosphate buffered saline (PBS, pH = 7.4), aliquoted and stored at −80 °C. Target cells were transduced with the resulting lentiviruses in the presence of 8 μg/mL polybrene (Sigma–Aldrich). At 24-h after transduction, viruses were removed and replaced with fresh media.

### Generation of mutant cell libraries and screening

A total of ~2 × 10^8^ PK-15-Cas9 cells were seeded into T225 flasks and infected with the library lentiviruses at an MOI of 0.3. Three days post-infection, GFP-positive cells were collected by Fluorescence-Activated Cell Sorting and reseeded into 100 mm dishes. Six days post-infection, DNA from ~7 × 10^6^ cells was extracted using a Blood & Cell Culture DNA Midi Kit (QIAGEN) and amplified to examine the coverage of mutant cell libraries. For the CRISPR screening, ~6 × 10^7^ mutant cells were infected with JEV-RP9 at an MOI of 0.03 in DMEM without FBS and incubated at 37 °C and 5% CO_2_. After 1.5-h incubation, the inoculum was removed and replaced with fresh DMEM supplementing with 2% FBS and 1% penicillin–streptomycin. At the eleventh day after post-infection, viable cells were collected and expanded for the deep sequencing analysis and the next round of infection.

### Knockout of candidate genes in PK-15 cells by CRISPR/Cas9

Individual sgRNA targeting to candidate gene was cloned into the linearized lenti-sgRNA-EGFP and lentivirus were produced as described above. The resulting lentivirus was transduced into PK-15-Cas9 cells. Cells with GFP expression were enriched by FACS and then seeded into 96-well plates to generate clonal knockout. At the seventh day after transduction, the genotypes of cell colonies were analyzed by extracting genomic DNA (TIANamp Genomic DNA Kit, TIANGEN) and sequencing. All primers for identifying the genotype of cell colonies are listed in Supplementary Data 4.

### Transfection of siRNAs

siRNAs were synthesized by RIBOBIO (Guangzhou, China). PK-15 and ST cells were seeded into 12-well plates and transfected (approximately 60% confluent) with 80 pmol of siRNA using Lipofectamine 2000 (Invitrogen) according to the manufacturer’s instructions. A negative control siRNA (siRNA-NC) was also transfected into cells as a negative control. At 48-h after transfection, the cells were incubated with JEV-RP9 at 37 °C with 5% CO_2_. At 2-h after incubation, inoculum was removed, and 2 mL of fresh medium with 10% FBS and 1% penicillin–streptomycin was added. At 18-h after incubation, the treatment cells were used for further functional analysis.

### Real-time reverse transcription PCR (qRT-PCR) analysis

Total RNA from cells was extracted with TransZol Up (Transgen), and viral RNAs were extracted from cell suspensions using a Viral RNA Extraction Kit (TaKaRa) following the manufacturer’s instructions, respectively. RNA was measured using a NanoDrop 2000 spectrophotometer (Thermo Scientific) for assessing RNA quantity and quality. cDNAs were synthesized using the PrimeScript™ RT reagent Kit with gDNA Eraser (TaKaRa). Then, cDNA products used as templates for relative quantitative real-time qPCR. The qPCR reactions were prepared with RealUniversal SYBR Green Premix (TIANGEN) following the manufacturer’s instructions. Briefly, PCR mixtures (10 µL) contained 5 µL RealUniversal Premix, 0.3 µL forward primer (0.3 µM), 0.3 µL reverse primer (0.3 µM), and 1 µL cDNA template. The results were monitored using a CFX384 Real-Time PCR Detection System (Bio-Rad, USA) programmed for one cycle of 15 min at 95 °C, followed by 39 cycles of 10 s at 95 °C, 30 s at 60 °C. Relative expression levels were calculated using the 2^−△△Ct^ method. The glyceraldehyde-3-phosphate dehydrogenase (GAPDH) gene was used as a normalization control. For absolute quantitative real-time PCR, about 1 µL of viral RNAs were used as template to synthesize cDNAs. Absolute quantitative real-time PCR assay was performed with SYBR green I (TOYOBO) and primers binding to *C* gene of JEV in a final reaction volume of 20 µL. PCR mixtures contained 10 µL SYBR® Green Real-time PCR Master Mix, 1 µL forward primer (0.5 µM), 1 µL reverse primer (0.5 µM), and 2 µL cDNA template. The results were monitored using a LightCycler® 96 System (Roche) programmed for one cycle of 15 min at 95 °C, followed by 45 cycles of 10 s at 95 °C, 10 s at 60 °C, 10 s at 72 °C. The JEV C protein encoding cDNA sequence from GenBank (accession number: AF014161.1) was cloned into pMD19-T vector and used as a standard for the quantification of JEV copy numbers. All primers used in quantitative PCR are listed in Supplementary Data 4.

### T7 endonuclease I cleavage detection assay and Sanger sequencing

All potential off-target sites with high homology in the sgRNAs were predicted using software CRISPR-offinder^26^. Genomic DNAs were extracted using the TIANamp Genomic DNA Kit (TIANGEN) from mutated clonal cells for PCR amplification, and T7 endonuclease I (T7EN I) cleavage detection assay was employed to determine off-target effects. CRISPR/Cas9-induced lesions at the endogenous target site and predicted off-targets were quantified using the T7EN I cleavage detection assay to investigate the insertions/deletions (indels) generated by nuclease-mediated non-homologous end joining (NHEJ). The gene fragments of off-target sites were amplified with primers specific to each locus by 35 cycles of PCR with TaKaRa LA Taq (TaKaRa). The PCR products were purified, denatured, and annealed using a thermocycler, and the hybridized PCR products were digested with T7EN I (NEB) for 15 min and separated with a 2% agarose gel. The agarose gels were stained with Gel-Red and the signal of DNA in the gel was quantified by densitometry using Image Lab software (Bio-Rad). All primers are listed in Supplementary Data 4.

### Illumina sequencing of sgRNAs in the genome-wide library and enriched mutants

The genomic DNA of each sample was extracted using a Blood & Cell Culture DNA Midi Kit (QIAGEN). The sgRNA-coding region was amplified by PCR using Q5® Hot Start High-Fidelity DNA Polymerase (NEB) in a reaction volume of 50 µL. PCR products were mixed and purified with a MinElute PCR purification Kit (QIAGEN). The purified PCR products were amplified by PCR using different barcoded primers. All PCR products were pooled and purified with a MinElute PCR purification Kit (QIAGEN), followed by Illumina HiSeq 3000 Next-generation sequencing. Mapped read counts were subsequently used as input for the MAGeCK analysis software package (version 0.5)^56^. Then, the top 0.5% ranked sgRNAs from the third and fourth JEV challenge rounds were used to identify enriched targeting protein-coding genes. Kyoto Encyclopedia of Genes and Genomes (KEGG) enrichment analyses were performed in the Database for Annotation, Visualization and Integrated Discovery (DAVID) (https://david.ncifcrf.gov/)^57^. All primers are listed in Supplementary Data 4.

### Virus plaque assay

Plaque assays were performed on BHK-21 cells. Briefly, BHK-21 cell monolayers at 50% confluence were incubated with serially diluted virus at 37 °C with 5% CO_2_. At 2 hrs after incubation, inoculum was removed, and the cells were overlaid with 50% 2× DMEM, 50% Agarose LMP (Genview), 2% FBS and 1% penicillin–streptomycin for 3 days. Cells were fixed with 10% formaldehyde neutral solution overnight at room temperature, then stained with 0.5% crystal violet for 2 h at room temperature. Plaques were counted manually and plaque-forming units were calculated. Three independent experiments were performed, with results presented as means ± S.D.

### Immunofluorescence assay

The expression level of JEV NS3 protein in WT or gene KO PK-15 cells as indicated in figures was determined by immunofluorescence assay. Briefly, cells grown on the glass coverslip in 6-well cell culture plates were infected with JEV at different MOI. After infection with different setting times, cells were fixed with 4% paraformaldehyde for 15 min at room temperature, washed twice with precooled PBS, and then permeabilized for 10 min at room temperature with cold 0.3% TritonX-100 in PBS. Cells were reacted with NS3 (JEV) antibody (GeneTex, #GTX125868, 1:5000) or anti-Heparin/Heparan Sulfate antibody (US Biological, 10E4, #H1890, 1:100) at 4 °C overnight, and the primary antibodies were recognized by Alexa Fluor® 594 Donkey anti-rabbit IgG (H + L) (Antgene, #ANT030, 1:400) or Alexa Fluor® 555 Anti-mouse IgG (H + L) (Cell Signaling Technology, #4409 S, 1:1000) and shaken at room temperature for 1.5 h in the dark. Cell nuclei were counter-stained with DAPI (4’, 6-diamidino-2-phenylindole) (Beyotime, #C1005) for 10 min at room temperature in the dark. Cells were observed with a fluorescence microscope (OLYMPUS IX3-RFACS), and images were taken with OLYMPUS DP80 camera. The proportion of NS3-positive cells were calculated by Image J software (three independent wells were imaged and one random field per well was captured for each experimental phase respectively).

### Immunoblotting assay

Approximately 1.2 × 10^6^ of PK-15 cells were lysed in ice-cold cell lysis buffer (50 mM Tris-HCl [pH = 6.8], 2% SDS, 10% glycerol, 1% β-mercaptoethanol, 12.5 mM EDTA, 0.02% bromophenol blue) supplemented with protease inhibitor (Beyotime) and phenylmethylsulfonyl fluoride (PMSF) (Beyotime), with cell lysates precleared at 4 °C by centrifugation at 15,871 × *g* for 10 min. The precleared lysates were separated by 10% polyacrylamide gel SDS. Separated proteins were then transferred onto a nitrocellulose membrane and probed with Cas9 (GeneTex, #GTX53807, 1:3000), EMC3 (Santa Cruz Biotechnology, #sc-365903, 1:500), CALR (Abclonal, #A1066, 1:1000) antibody, with β-tubulin (Sungenebiotech, #KM9003T, 1:5000)/GAPDH antibodies (Beyotime, #AF5009, 1:3000) used as an internal loading control. The primary antibodies were detected with horseradish peroxidase (HRP) conjugated goat anti-rabbit IgG (Abclonal, #AS014, 1:5000) or goat anti-mouse IgG (Beyotime, A0216, 1:1000), and the secondary antibodies were visualized by ECL Prime Western Blotting Detection Reagents (GE Healthcare, UK).

### Real-time cell analysis

The real-time dynamic monitoring of CPE was performed on WT or KO PK-15 cell cultures infected with the JEV using RTCA xCELLigence System (Roche, Basel. Switzerland). First, 50 µL of DMEM medium with 10% FBS, 100 U/mL penicillin and 100 μg/mL streptomycin were added to each well for background measurement. Then, about 4000 WT or KO cells were seeded in a 96-well E-plate®. The E-plate® was incubated at room temperature for 30 min and placed on the reader in the incubator for continuous recording of impedance to compute the cell index (CI). At 0 h after incubation, the cells were infected with JEV at an MOI of 0.03 or 1. The impedance-based CI was quantified by RTCA software program version 1.2.1 (Roche, Basel. Switzerland), data was gathered at 15-min intervals for 60 or 90 h at 37 °C in a 5% CO_2_ humidified atmosphere, and normalized according to the last time point prior the start of treatment (t = 0 h). From this data, the real-time cell growth curves were generated with GraphPad Prism 7 (GraphPad Software, La Jolla, CA, USA).

### EdU cell proliferation assay

To assess cell proliferation, KO and WT PK-15 cells were seeded into 12-well plates. The cells were maintained in DMEM medium with 10% FBS, 100 U/mL penicillin and 100 μg/mL streptomycin, which incubated at 37 °C with 5% CO_2_. At 24-h after incubation, EdU cell proliferation assays were performed with the BeyoClick™ EdU Cell Proliferation Kit with Alexa Fluor 555 (Beyotime, #C0075S) according to instruction. The cell nuclei were stained with DAPI (Beyotime, #C1005) at room temperature for 10 min at room temperature in the dark. Stained cells were visualized under a fluorescence microscope. The proportion of EdU-positive cells were calculated by Image J software (three independent wells were imaged and one random field per well was captured for each experimental phase, respectively).

### Transmission electron microscopy

Approximately 1 × 10^7^ of *CALR* KO, *EMC3* KO, and WT PK-15 cells were infected with JEV-RP9 at an MOI of 1 for 24 h. The cells were washed twice with precooled PBS and fixed by adding 3 mL of fixative (Servicebio) for 2 h at room temperature. After fixation, cells were scraped and transferred into 15 mL centrifuge tube and centrifuged at 251.55 × *g* for 5 min at 4 °C. Negative-staining electron microscopy and image analysis process was performed by Servicebio company.

### Statistical analysis

Statistical analysis was performed using R programming language. The means ± S.D. was determined for each treatment group in the separated experiments. Two-tailed Student’s *t*-test was used to determine significant differences between treatment and control groups (*P < 0.05; **P < 0.01; ***P < 0.001; ****P < 0.0001, ns no significant).

### Reporting summary

Further information on research design is available in the Nature Research Reporting Summary linked to this article.

## Supplementary information

Supplementary Information

Description of Additional Supplementary Files

Supplementary Data 1

Supplementary Data 2

Supplementary Data 3

Supplementary Data 4

Reporting Summary

## Data Availability

Raw sequencing data and processed counts data for sgRNA libraries that support the findings of this study have been deposited in National Center for Biotechnology Information’s Gene Expression Omnibus and are accessible through GEO Series accession number GSE158247. All other relevant data supporting the key findings of this study are available within the article and its Supplementary Information files, and can also be requested from the corresponding authors. Supplemental Information and Supplementary Data files can be found with this article online. Source data are provided with this paper.

## Source data

Source Data
