# Supplementary material for: CRISPR screening of porcine sgRNA library identifies host factors associated with Japanese encephalitis virus replication
Source: Nat Commun. 2020 Oct 14;11:5178. doi: 10.1038/s41467-020-18936-1 (PMC7560704; doi:10.1038/s41467-020-18936-1)
Supplement: Supplementary file 1 — Supplementary Information [file 41467_2020_18936_MOESM1_ESM.doc]

**Supplemental Information**

**CRISPR screening of** **porcine sgRNA library identifies** **host factors associated with Japanese encephalitis virus replication**

Changzhi Zhao^1#^, Hailong Liu^1#^, Tianhe Xiao^1#^, Zichang Wang^1^, Xiongwei Nie^1^, Xinyun Li^1, 3^, Ping Qian^2, 3^, Liuxing Qin^2^, Xiaosong Han^1^, Jinfu Zhang^1^, Jinxue Ruan^1^, Mengjin Zhu^1, 3^, Yiliang Miao^1, 3^, Bo Zuo^1, 3^, Kui Yang^4^, Shengsong Xie^1, 3^*, Shuhong Zhao^1, 3^*

^1^Key Laboratory of Agricultural Animal Genetics, Breeding and Reproduction of Ministry of Education & Key Lab of Swine Genetics and Breeding of Ministry of Agriculture and Rural Affairs, Huazhong Agricultural University, Wuhan 430070, P. R. China;

^2^State Key Laboratory of Agriculture Microbiology, Huazhong Agricultural University, Wuhan 430070, P. R. China;

^3^The Cooperative Innovation Center for Sustainable Pig Production, Huazhong Agricultural University, Wuhan 430070, P. R. China;

^4^Louisiana State University, School of Veterinary Medicine, Baton Rouge, Louisiana 70803, USA.

*To whom correspondence should be addressed. Shuhong Zhao, Huazhong Agricultural University, No.1, Shizishan Street, Hongshan District, Wuhan, Hubei Province, 430070, P. R. China. Tel: 086-027-87387480; Fax: 086-027-87280408; Email: shzhao@mail.hzau.edu.cn;

Correspondence may also be addressed to Shengsong Xie, Huazhong Agricultural University, No.1, Shizishan Street, Hongshan District, Wuhan, Hubei Province, 430070, P. R. China. Tel: 086-027-87387480; Fax: 086-027-87280408; Email: ssxie@mail.hzau.edu.cn.

^#^The authors contributed equally.

**Table of Contents**

[Supplementary Fig. 1 Generation of a single cell–derived Cas9-expression clone. 3](#__RefHeading___Toc18853)

[Supplementary Fig. 2 Evaluation of the cleavage efficiency of Cas9 in PK-15-Cas9 cells. 4](#__RefHeading___Toc5527)

[Supplementary Fig. 3 Evaluation of off-target effects of a randomly selected sgRNA (targeting the IZUMO3 gene) from originally designed sgRNA library. 5](#__RefHeading___Toc13775)

[Supplementary Fig. 4 Determine the optimal infection dose of JEV-induced PK-15 cell death. 6](#__RefHeading___Toc30376)

[Supplementary Fig. 5 Over-expression of a scrambled sequence negative control sgRNA (sgRNA-NC) in PK-15 cells did not affect JEV replication. 6](#__RefHeading___Toc1675)

[Supplementary Fig. 6 Dynamic monitoring of wild-type and knockout cells during JEV infection by Real-Time Cell Analyzer assay. 8](#__RefHeading___Toc31435)

[Supplementary Fig. 7 Knockout of SLC35B2, HS6ST1, B3GAT3 or GLCE does not affect the proliferation of PK-15 cells. 9](#__RefHeading___Toc8383)

[Supplementary Fig. 8 Immunofluorescence for detection of NS3 protein expressed in clonal SLC35B2, HS6ST1, B3GAT3 and GLCE knockout cell lines following infection with JEV. 10](#__RefHeading___Toc7027)

[Supplementary Fig. 9 Knockout of SLC35B3 or B3GAT3 results in hindrance of the plasmid delivery by lipofection transfection reagent. 10](#__RefHeading___Toc18329)

[Supplementary Fig. 10 Rescue assays for ectopic expression of HS6ST1 or GLCE in corresponding knockout cells resulted in partial recovery of JEV replication. 11](#__RefHeading___Toc2197)

[Supplementary Fig. 11 Knockout of HS6ST1 or GLCE can fully support viral replication by infectious JEV cDNA clone system. 12](#__RefHeading___Toc26312)

[Supplementary Fig. 12 Single knockdown of HSPG pathway genes coding SLC35B2, HS6ST1, B3GAT3, and GLCE proteins led to significant inhibition of JEV replication at an MOI of 0.1 in PK-15 or ST cells. 13](#__RefHeading___Toc21609)

[Supplementary Fig. 13 Single knockdown of HSPG pathway genes coding SLC35B2, HS6ST1, B3GAT3, and GLCE proteins led to significant inhibition of JEV replication at an MOI of 0.03 in PK-15 or ST cells. 14](#__RefHeading___Toc28617)

[Supplementary Fig. 14 Double knockdown of HSPG pathway genes coding SLC35B2, HS6ST1, B3GAT3, and GLCE proteins led to significant inhibition of JEV replication at an MOI of 0.1 in PK-15 cells. 15](#__RefHeading___Toc14870)

[Supplementary Fig. 15 Knockout of EMC3 or CALR does not affect the proliferation of PK-15 cells. 16](#__RefHeading___Toc27218)

[Supplementary Fig. 16 Rescue assays for ectopic expression of EMC3 or CALR in corresponding knockout cells resulted in partial recovery of JEV replication. 17](#__RefHeading___Toc16097)

[Supplementary Fig. 17 Knockdown of EMC3 or CALR led to significant inhibition of JEV replication at an MOI of 0.1 in PK-15 or ST cells. 18](#__RefHeading___Toc6497)

[Supplementary Fig. 18 Knockdown of EMC3 or CALR led to significant inhibition of JEV replication at an MOI of 1 in PK-15 or ST cells. 19](#__RefHeading___Toc10635)

[Supplementary Fig. 19 Observation of virus particle assembly and ER morphology in EMC3-deficient cells by negative-staining electron microscopy. 21](#__RefHeading___Toc24956)

[Supplementary Fig. 20 Observation of virus particle assembly and mitochondrial morphology in CALR-deficient cells by negative-staining electron microscopy 22](#__RefHeading___Toc27150)

**Supplementary Figures and legends**

**
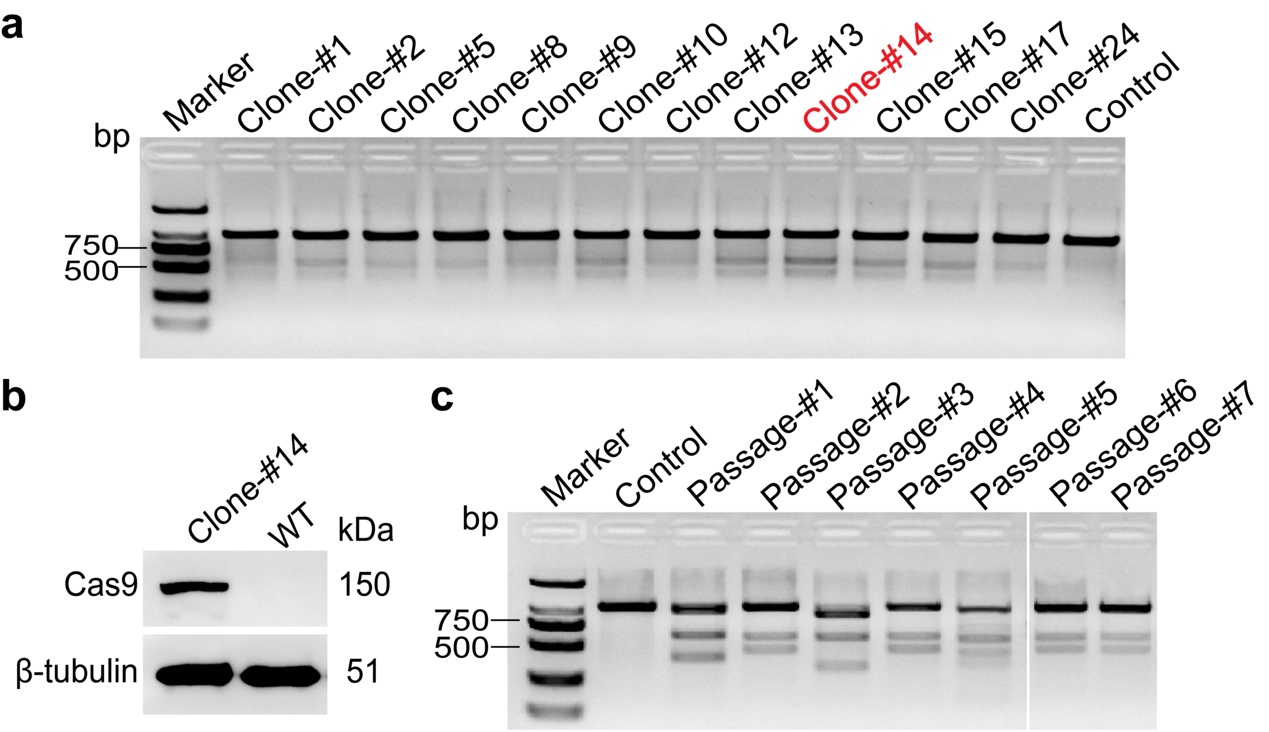
**

**Supplementary Fig. 1 Generation of a single cell–derived Cas9-expression clone. a**, Evaluation of Cas9 activity among candidate single cell–derived clones using a T7EN I cleavage assay. The candidate cells were transduced with a validated sgRNA (targeting the *ANPEP* gene) lentivirus. The single cell–derived clone with the highest Cas9 activity (Clone-#14, PK-15-Cas9) is indicated in red. **b**, Western blot was performed to examine the expression of the Cas9 protein in the single cell–derived Clone-#14. β-tubulin was used as loading control. **c**, Evaluation of the stability and activity of Cas9 in the resulting single cell–derived clone (Clone-#14, PK-15-Cas9) using a T7EN I cleavage assay. The candidate cells were transduced with the same sgRNA lentivirus as (**a**). Passage-#1-#7: cell passage number. bp: base pairs; kDa: Kilodalton; Control: wild-type cells. Marker: DL2000 DNA ladder. The experiments were repeated two times with similar results and representative results shown (**b**). Source data are provided as a Source Data file.

**
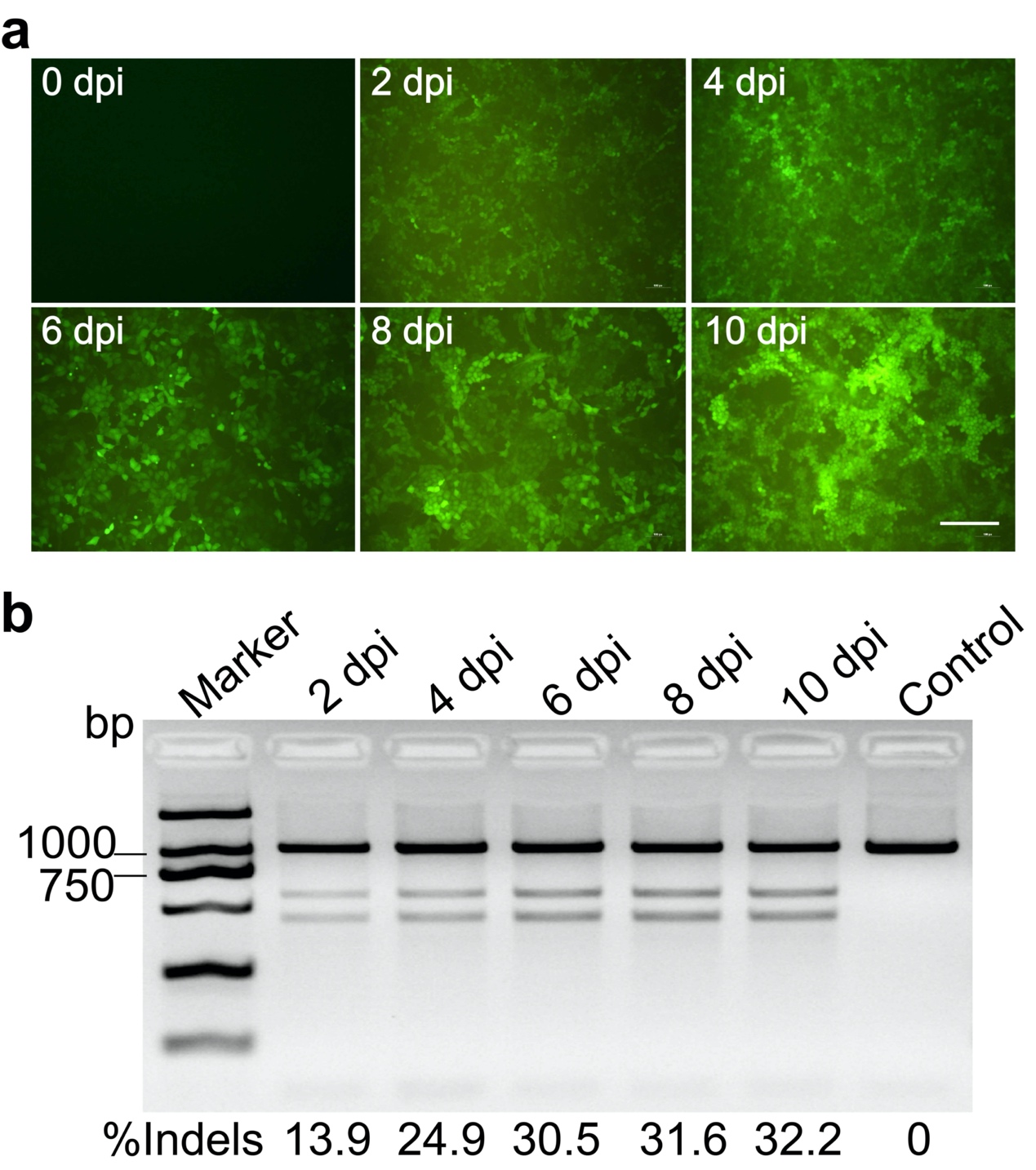
**

**Supplementary Fig. 2 Evaluation of the cleavage efficiency of Cas9 in PK-15-Cas9 cells. a**, Assessment of the transduction efficiency of sgRNA lentivirus in PK-15-Cas9 cells at the time points indicated. **b**, Assessment of the cleavage activity of sgRNA lentivirus in PK-15-Cas9 cells at the time points indicated using a T7EN I assay. %Indels: percentage of indels; bp: base pairs; dpi: days post infection, Control: wild-type cells; Marker: DL2000 DNA ladder. Scale bar, 200 μm. The experiments were repeated two times with similar results and representative results shown (**b**). Source data are provided as a Source Data file.

**
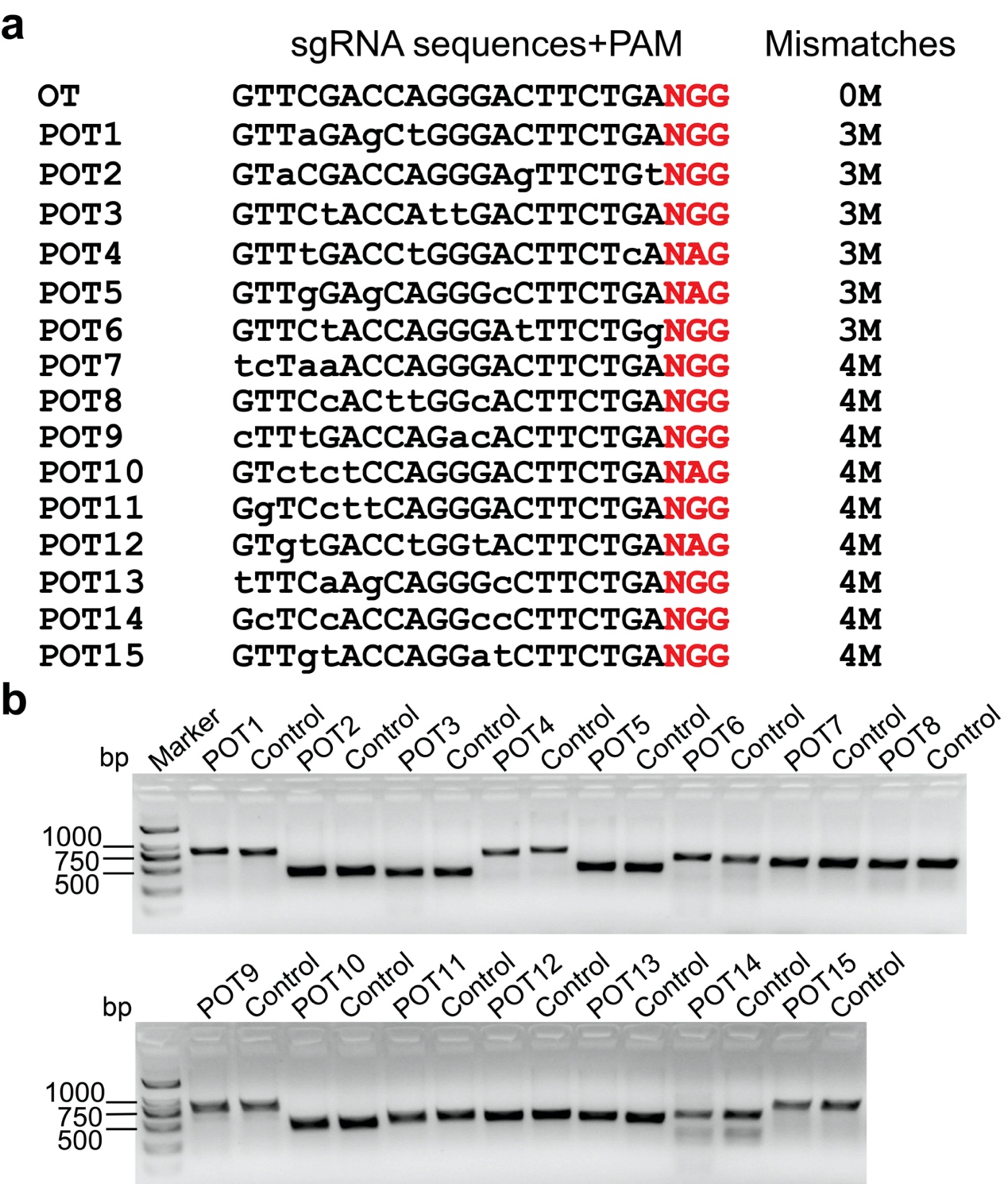
**

**Supplementary Fig. 3 Evaluation of off-target effects of a randomly selected sgRNA (targeting the IZUMO3 gene) from originally designed sgRNA library. a**, Prediction of potential off-targets of candidates sgRNA by CRISPR-offinder. Mismatched nucleotide letters are indicated in lowercase. PAM sites are indicated in red letters, N stands for A, T, C or G. **b**, Detection of the 15 potential off-targets by the T7EN I cleavage assay. PAM: protospacer adjacent motif; sgRNA: small guide RNA; OT: on-target; POT: Potential off-target effect; Control: wild-type cells; bp: base pairs. M, the number of nucleotide mismatches (1M, 2M, 3M or 4M); 0M, perfect match to the on-target site. Source data are provided as a Source Data file.


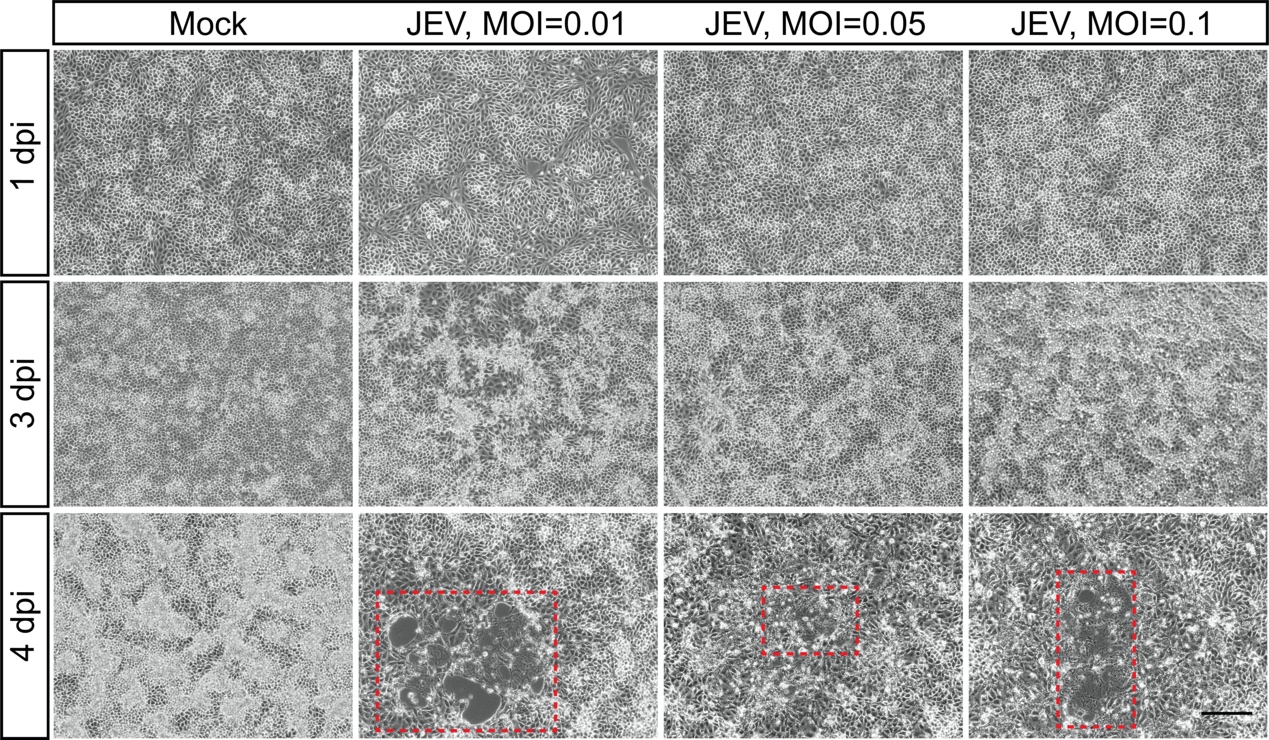


**Supplementary Fig. 4 Determine the optimal infection dose of JEV-induced PK-15 cell death.** The red box indicates JEV-induced cytopathic effects (CPE) in PK-15 cells infected with JEV at MOIs of 0.01, 0.05 or 0.1. Mock: non-infected cells was included as negative control; JEV: Japanese Encephalitis Virus; MOI: multiplicity of infection; dpi: days post-infection. Scale bar, 200 μm.


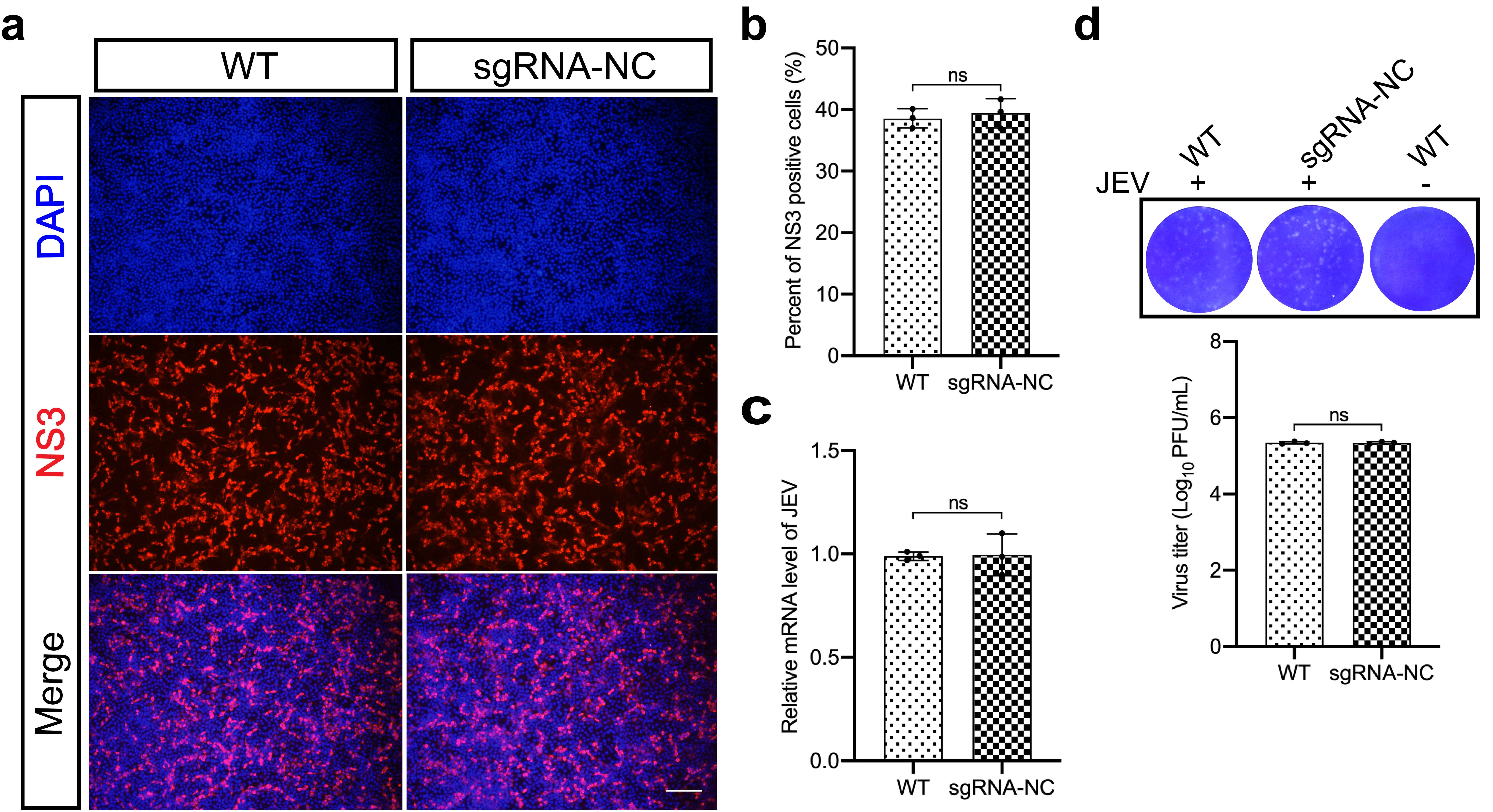


**Supplementary Fig. 5 Over-expression of a scrambled sequence negative control sgRNA (sgRNA-NC) in PK-15 cells did not affect JEV replication. a**, **Detection** of NS3 protein by immunofluorescence in a sgRNA-NC stable cell line following infection with JEV at an MOI of 1. Scale bar, 200 μm. **b**, There was no significant difference in the proportion of NS3 positive cells in a sgRNA-NC stable cell line as compared with WT cells. Quantitative analysis of Immunofluorescence (**a**) was used by Image J software. **c**, RT-qPCR assay for determination of relative mRNA level of JEV *C* gene in a sgRNA-NC stable cell line following infection with JEV at an MOI of 1. *GAPDH* was used as a reference gene. **d**, Virus plaque assays for determination of viral concentration in a sgRNA-NC stable cell line following infection with JEV at an MOI of 1. WT: wild-type cells (or untreated cells), sgRNA-NC: a scrambled sequence negative control sgRNA (negative control); DAPI: 4′, 6-diamidino-2-phenylindole. Data are represented as means ± S.D.; n = 3 (**b, c, d**). ns: no significant. *P*-values were determined by two-sided Student’s t-test. Source data are provided as a Source Data file.





**Supplementary Fig. 6 Dynamic monitoring of wild-type and knockout cells during JEV infection by Real-Time Cell Analyzer assay.** *SLC35B2*, *HS6ST1*, *B3GAT3*, *GLCE* KO cell lines were infected with JEV at an MOI of 0.03 (**a**) or 1 (**b**), respectively. Cell index values reflect the biological statue of monitored cells, including the cell number, cell viability, morphology and adhesion degree. Real-time monitoring of cell proliferation measured as cell index was recorded every 15 min for up to 90 hrs (**a**) or 60 hrs (**b**) after cell lines were infected with JEV. Data represent changes in cell index over time. Mock: non-infected cells was included as negative control; WT: wild-type cells; KO: knockout cells; JEV: Japanese Encephalitis Virus; MOI: multiplicity of infection; hpi: hours post-infection. Colored curves indicate the WT and KO cell lines with or without JEV infection seeded per well in an E-Plate 96.


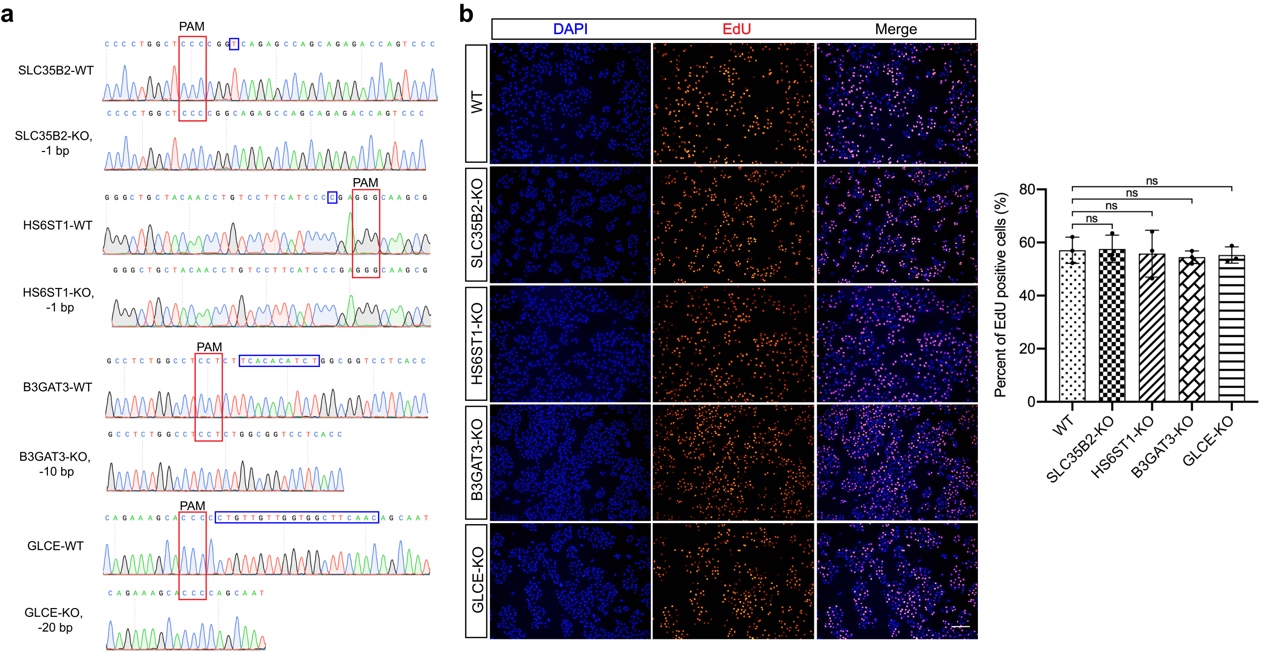


**Supplementary Fig. 7 Knockout of SLC35B2, HS6ST1, B3GAT3 or GLCE does not affect the proliferation of PK-15 cells. a,** DNA sequence analysis showed the presence of the mutation in clonal KO cells of *SLC35B2*, *HS6ST1*, *B3GAT3*, and *GLCE*. The blue box indicates the deleted bases in the KO cells. The red box indicates the PAM sites. **b,** There was no significant change in the proportion of EdU positive cells in clonal *SLC35B2*, *HS6ST1*, *B3GAT3*, and *GLCE* KO cell lines as compared with WT cells. Left: representative pictures. Right: quantification of EdU positive cells. Quantitative analysis of Immunofluorescence was used by Image J software. PAM: protospacer adjacent motif; WT: wild-type cells; KO: knockout cells; DAPI: 4′, 6-diamidino-2-phenylindole. Scale bar, 200 μm. Data are represented as means ± S.D.; n = 3 (**b**). ns: no significant. *P*-values were determined by two-sided Student’s t-test. Source data are provided as a Source Data file.

**
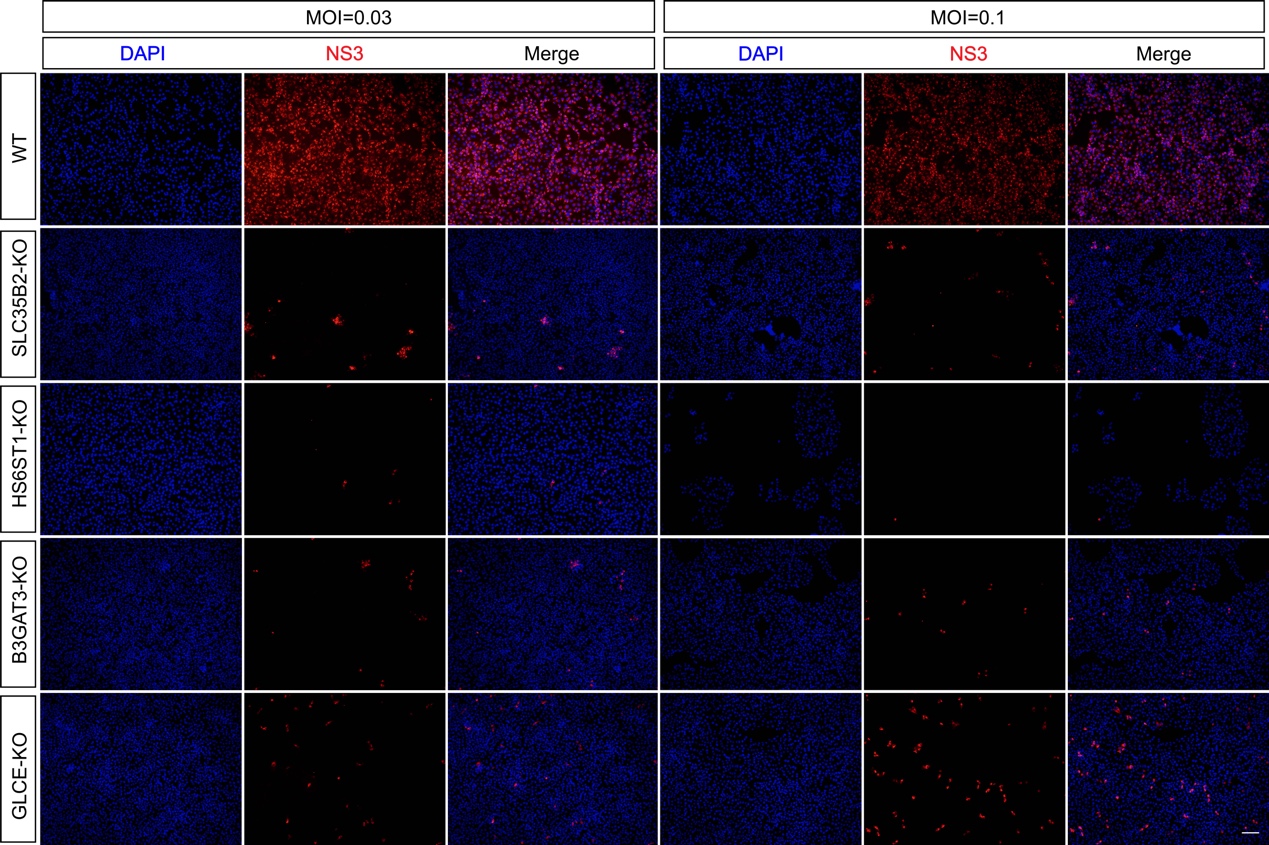
**

**Supplementary Fig. 8 Immunofluorescence for detection of NS3 protein expressed in clonal SLC35B2, HS6ST1, B3GAT3 and GLCE knockout cell lines following infection with JEV.** WT: wild-type cells; KO: knockout cells; MOI: multiplicity of infection; DAPI: 4′, 6-diamidino-2-phenylindole. Scale bar, 200 μm.


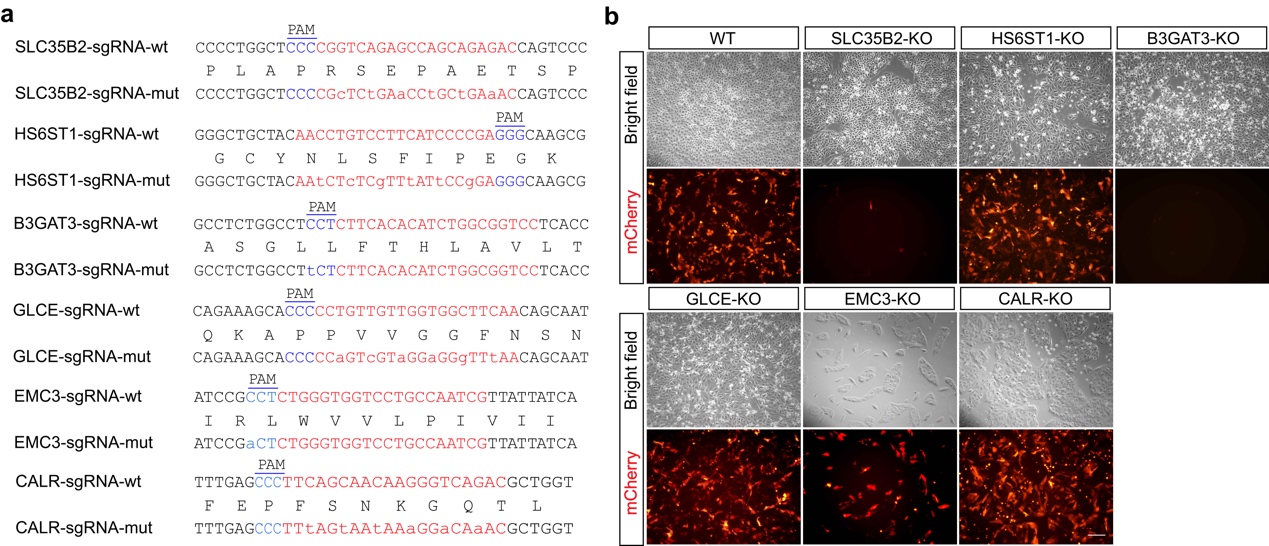


**Supplementary Fig. 9 Knockout of SLC35B3 or B3GAT3 results in hindrance of the plasmid delivery by lipofection transfection reagent. a**, Schematic illustration of the target sequences + PAM sequence (in blue letters) into *SLC35B2*, *HS6ST*1, *B3GAT3*, *GLCE*, *EMC3* and *CALR* loci. sgRNA targeting sites are highlighted in red, small letters mutations introduced into the genome. These mutations prevent further cleavage of mutated target genes by CRISPR/Cas9 technology. **b**, Transfection of mCherry fluorescent protein plasmid into corresponding KO cells by Lipofectamine 2000 reagent. PAM: protospacer adjacent motif; wt: wild-type gene; mut: mutation; WT: wild-type cells; KO: knockout cells; mCherry: (pmCherry-N1 plasmid, TAKARA). Scale bar, 200 μm.





**Supplementary Fig. 10 Rescue assays for ectopic expression of HS6ST1 or GLCE in corresponding knockout cells resulted in partial recovery of JEV replication. a**, Detection of NS3 protein by Immunofluorescence. The proportion of NS3 positive cells was significantly increased during restoration of HS6ST1 expression in *HS6ST1* KO cells (shown as in Figure 4. e). **b**, RT-qPCR assay for determination of relative mRNA level of *HS6ST1*. **c**, Detection of NS3 protein by Immunofluorescence. The proportion of NS3 positive cells was significantly increased during restoration of GLCE expression in *GLCE* KO cells (shown as in Figure 4. g). **d**, RT-qPCR assay for determination of relative mRNA level of *GLCE*. WT: wild-type cells; KO: knockout cells; DAPI: 4′, 6-diamidino-2-phenylindole; HS6ST1-KO-rescue: Transfection of pcDNA3.1-HS6ST1 vector in clonal KO cell of *HS6ST1*; HS6ST1-KO-NTC: Transfection of pcDNA3.1 empty vector in clonal KO cell of *HS6ST1*. GLCE-KO-rescue: Transfection of pcDNA3.1-GLCE vector in clonal KO cell of *GLCE*; GLCE-KO-NTC: Transfection of pcDNA3.1 empty vector in clonal KO cell of *GLCE*. Scale bar, 200 μm. Data are represented as means ± S.D.; n = 3 (**b** and **d**). ** *P* < 0.01; ns: no significant. *P*-values were determined by two-sided Student’s t-test. Source data are provided as a Source Data file.


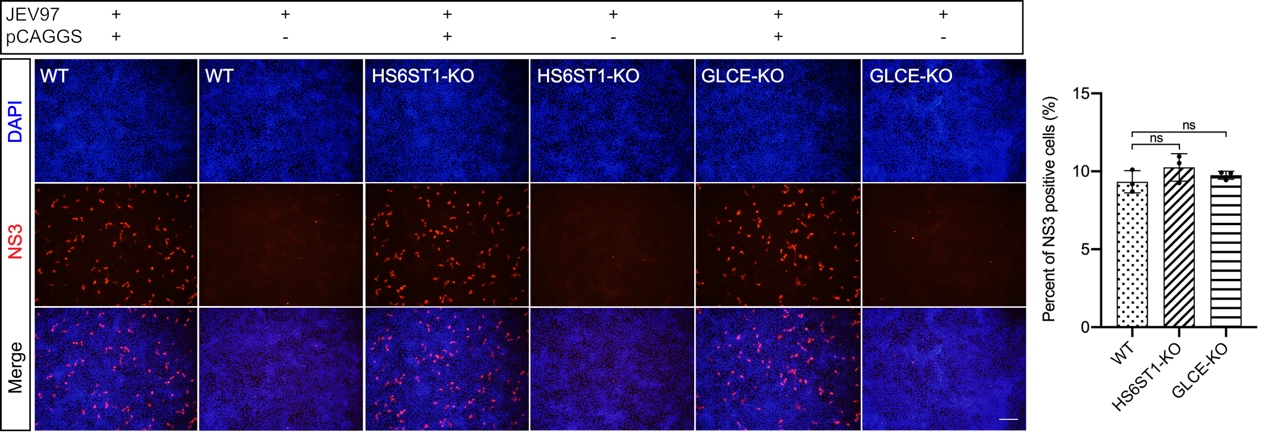


**Supplementary Fig. 11 Knockout of HS6ST1 or GLCE can fully support viral replication by infectious JEV cDNA clone system.** The full-length cDNA copy of the JEV genome was cloned into JEV97 vector under the control of T7 promoter. T7 RNA polymerase was expressed by pCAGGS vector. Detection of NS3 protein by Immunofluorescence. Left: representative pictures. Right: quantification of NS3 positive cells. WT: wild-type cells; KO: knockout cells; DAPI: 4′, 6-diamidino-2-phenylindole. Scale bar, 200 μm. Data are represented as means ± S.D.; n = 3. ns: no significant. *P*-values were determined by two-sided Student’s t-test. Source data are provided as a Source Data file.


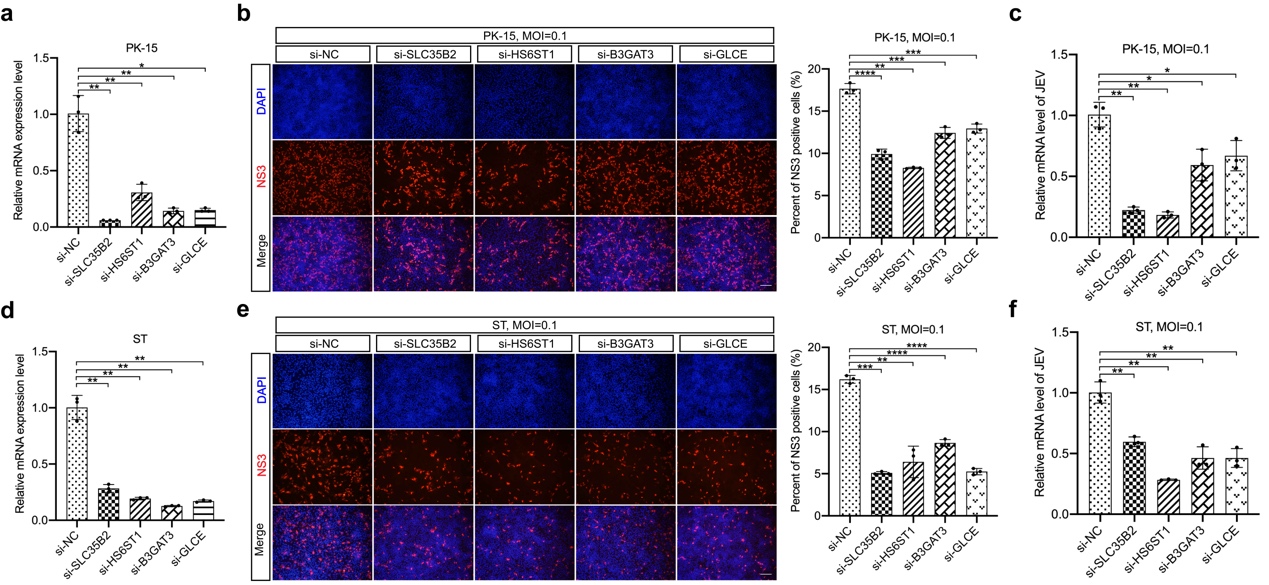


**Supplementary Fig. 12 Single knockdown of HSPG pathway genes coding SLC35B2, HS6ST1, B3GAT3, and GLCE proteins led to significant inhibition of JEV replication at an MOI of 0.1 in PK-15 or ST cells. a**, RT-qPCR assay for determination of relative mRNA level in *SLC35B2*, *HS6ST1*, *B3GAT3*, and *GLCE* knockdown cells as compared with control PK-15 cells. **b**, Detection of NS3 protein by Immunofluorescence. The proportion of NS3 positive cells was significantly decrease in *SLC35B2*, *HS6ST1*, *B3GAT3*, and *GLCE* knockdown cells as compared with control PK-15 cells. Left: representative pictures. Right: quantification of NS3 positive cells. **c**, RT-qPCR assay for determination of relative mRNA level of JEV *C* gene in *SLC35B2*, *HS6ST1*, *B3GAT3*, and *GLCE* knockdown cells following infection with JEV at an MOI of 0.1 (PK-15 cells). *GAPDH* was used as a reference gene. **d**, RT-qPCR assay for determination of relative mRNA level in *SLC35B2*, *HS6ST1*, *B3GAT3*, and *GLCE* knockdown cells as compared with control ST cells. **e**, Detection of NS3 protein by Immunofluorescence. The proportion of NS3 positive cells was significantly decrease in *SLC35B2*, *HS6ST1*, *B3GAT3*, and *GLCE* knockdown cells as compared with control ST cells. Left: representative pictures. Right: quantification of NS3 positive cells. **f**, RT-qPCR assay for determination of relative mRNA level of JEV *C* gene in *SLC35B2*, *HS6ST1*, *B3GAT3*, and *GLCE* knockdown cells following infection with JEV at an MOI of 0.1 (ST cells). *GAPDH* was used as a reference gene. si-NC: negative control siRNA; si-#(target): siRNA (knockdown cells); MOI: multiplicity of infection; DAPI: 4′, 6-diamidino-2-phenylindole; ST: a normal Sus scrofa testis-derived cell line; Quantitative analysis of Immunofluorescence (**b** and **e**) was used by Image J software. Scale bar, 200 μm. Data are represented as means ± S.D.; n = 3 (**a, b, c, d, e, f**). * *P* < 0.05; ** *P*< 0.01; *** *P* < 0.001; **** *P* < 0.0001. *P*-values were determined by two-sided Student’s t-test. Source data are provided as a Source Data file.


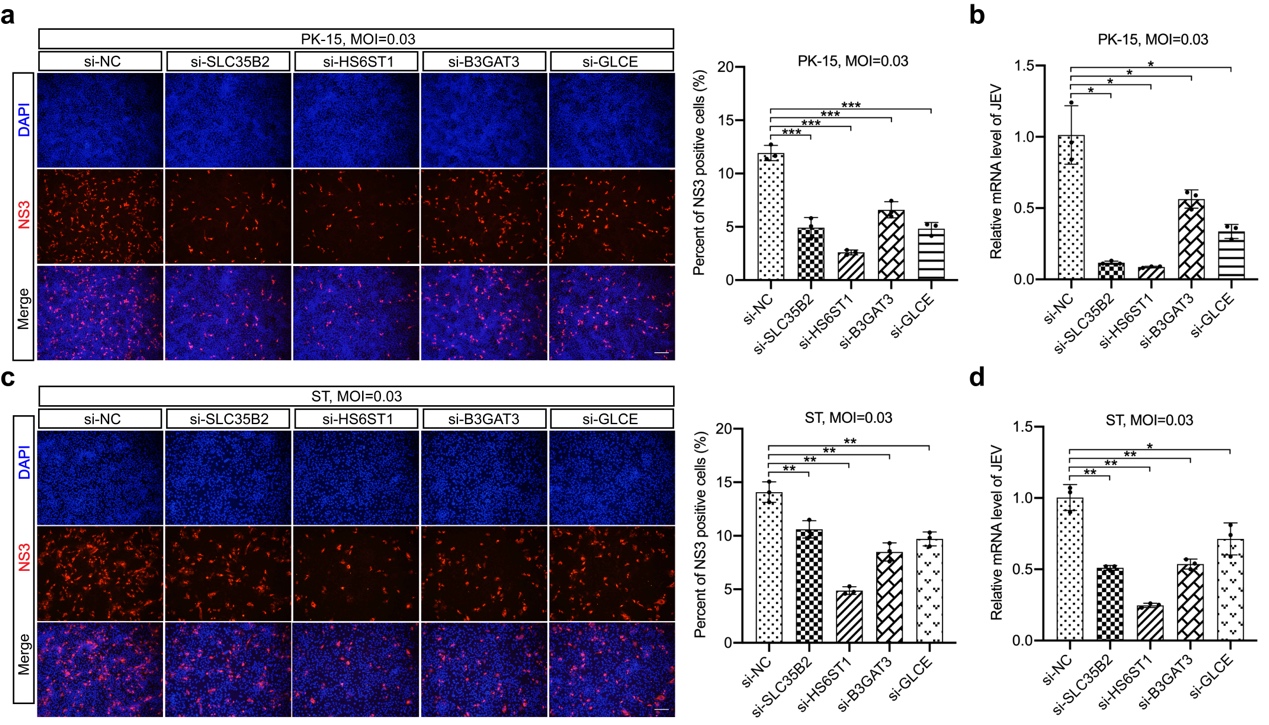


**Supplementary Fig. 13 Single knockdown of HSPG pathway genes coding SLC35B2, HS6ST1, B3GAT3, and GLCE proteins led to significant inhibition of JEV replication at an MOI of 0.03 in PK-15 or ST cells. a**, Detection of NS3 protein by Immunofluorescence. The proportion of NS3 positive cells was significantly decrease in *SLC35B2*, *HS6ST1*, *B3GAT3*, and *GLCE* knockdown cells as compared with control PK-15 cells. Left: representative pictures. Right: quantification of NS3 positive cells. **b**, RT-qPCR assay for determination of relative mRNA level of JEV *C* gene in *SLC35B2*, *HS6ST1*, *B3GAT3*, and *GLCE* knockdown cells following infection with JEV at an MOI of 0.03 (PK-15 cells). *GAPDH* was used as a reference gene. **c**, Detection of NS3 protein by Immunofluorescence. The proportion of NS3 positive cells was significantly decreased in *SLC35B2*, *HS6ST1*, *B3GAT3*, and *GLCE* knockdown cells as compared with control ST cells. Left: representative pictures. Right: quantification of NS3 positive cells. **d**, RT-qPCR assay for determination of relative mRNA level of JEV *C* gene in *SLC35B2*, *HS6ST1*, *B3GAT3*, and *GLCE* knockdown cells following infection with JEV at an MOI of 0.03 (ST cells). *GAPDH* was used as a reference gene. si-NC: negative control siRNA; si-#(target): siRNA (knockdown cells); MOI: multiplicity of infection; DAPI: 4′, 6-diamidino-2-phenylindole; ST: a normal Sus scrofa testis-derived cell line; Quantitative analysis of Immunofluorescence (**a** and **c**) was used by Image J software. Scale bar, 200 μm. Data are represented as means ± S.D.; n = 3 (**a, b, c, d**). * *P* < 0.05; ** *P* < 0.01; *** *P* < 0.001. *P*-values were determined by two-sided Student’s t-test. Source data are provided as a Source Data file.


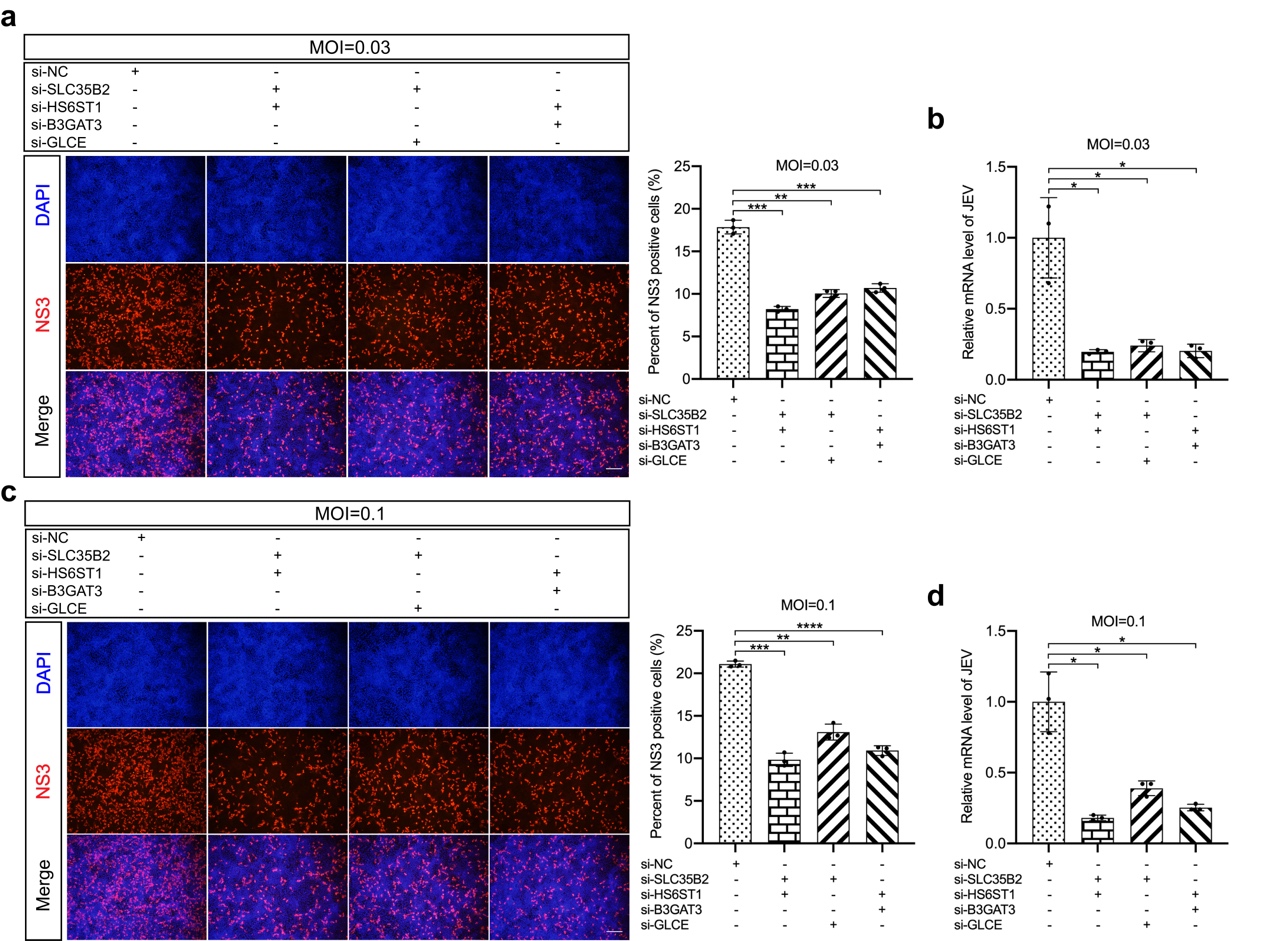


**Supplementary Fig. 14 Double knockdown of HSPG pathway genes coding SLC35B2, HS6ST1, B3GAT3, and GLCE proteins led to significant inhibition of JEV replication at an MOI of 0.03 or 0.1 in PK-15 cells. a**, Detection of NS3 protein by Immunofluorescence. The proportion of NS3 positive cells (MOI = 0.03) was significantly decrease in candidate genes double knockdown cells as compared with control PK-15 cells. Left: representative pictures. Right: quantification of NS3 positive cells. **b**, RT-qPCR assay for determination of relative mRNA level of JEV *C* gene in candidate genes double knockdown cells following infection with JEV at an MOI of 0.03. *GAPDH* was used as a reference gene. **c**, Detection of NS3 protein by Immunofluorescence. The proportion of NS3 positive cells (MOI = 0.1) was significantly decreased in candidate genes double knockdown cells as compared with control PK-15 cells. Left: representative pictures. Right: quantification of NS3 positive cells. **d**, RT-qPCR assay for determination of relative mRNA level of JEV *C* gene in candidate genes double knockdown cells following infection with JEV at an MOI of 0.1. *GAPDH* was used as a reference gene. si-NC: negative control siRNA; si-#(target): siRNA (knockdown cells); MOI: multiplicity of infection; DAPI: 4′, 6-diamidino-2-phenylindole; Quantitative analysis of Immunofluorescence (**a** and **c**) was used by Image J software. Scale bar, 200 μm. Data are represented as means ± S.D.; n = 3 (**a, b, c, d**). * *P* < 0.05; ** *P* < 0.01; *** *P* < 0.001; **** *P* < 0.0001. *P*-values were determined by two-sided Student’s t-test. Source data are provided as a Source Data file.





**Supplementary Fig. 15 Knockout of EMC3 or CALR does not affect the proliferation of PK-15 cells. a,** DNA sequence analysis showed the presence of the mutation in a clonal KO cell of *EMC3* or *CALR*. The blue box indicates the deleted/inserted bases in the KO cells. The red box indicates the PAM sites. **b,** The proportion of EdU positive cells was not significantly changed in a clonal *EMC3* or *CALR* KO cell line as compared with WT cells. Left: representative pictures. Right: quantification of EdU positive cells. Quantitative analysis of Immunofluorescence was conducted using Image J software. WT: wild-type cells; KO: knockout cells; DAPI: 4′, 6-diamidino-2-phenylindole. Scale bar, 200 μm. Data are represented as means ± S.D.; n = 3 (**b**). ns: no significant. *P*-values were determined by two-sided Student’s t-test. Source data are provided as a Source Data file.

**
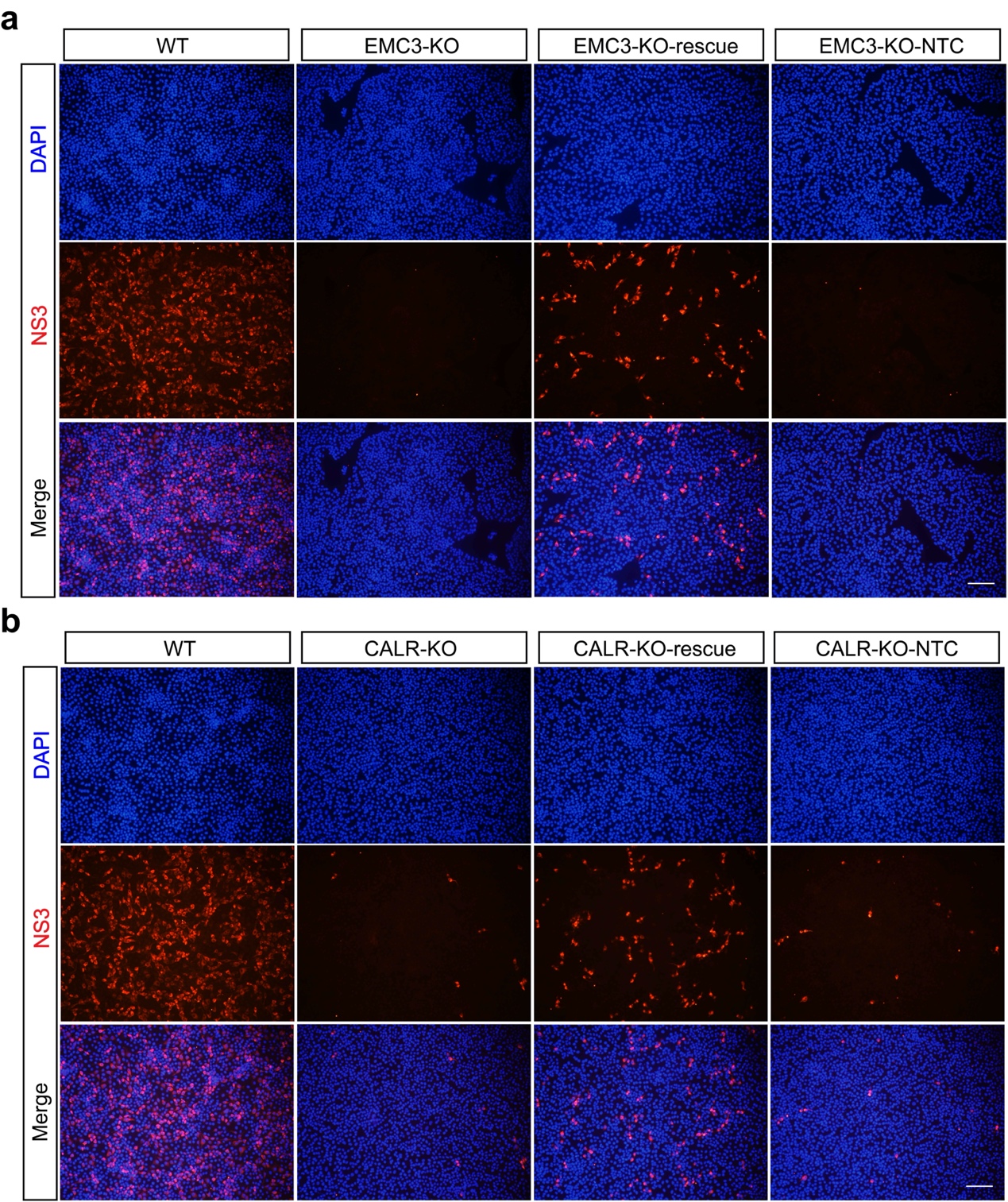
**

**Supplementary Fig. 16 Rescue assays for ectopic expression of EMC3 or CALR in corresponding knockout cells resulted in partial recovery of JEV replication. a**, Detection of NS3 protein by Immunofluorescence. The proportion of NS3 positive cells was significantly increased during restoration of EMC3 expression in *EMC3* KO cells (shown as in Figure 5. i). **b**, Detection of NS3 protein by Immunofluorescence. The proportion of NS3 positive cells was significantly increased during restoration of CALR expression in *CALR* KO cells (shown as in Figure 6. h). WT: wild-type cells; KO: knockout cells; DAPI: 4′, 6-diamidino-2-phenylindole; EMC3-KO-rescue: Transfection of pcDNA3.1-EMC3 vector in clonal KO cell of *EMC3*; EMC3-KO-NTC: Transfection of pcDNA3.1 empty vector in clonal KO cell of *EMC3*. CALR-KO-rescue: Transfection of pcDNA3.1-CALR vector in clonal KO cell of *CALR*; CALR-KO-NTC: Transfection of pcDNA3.1 empty vector in clonal KO cell of *CALR*. Scale bar, 200 μm.

**
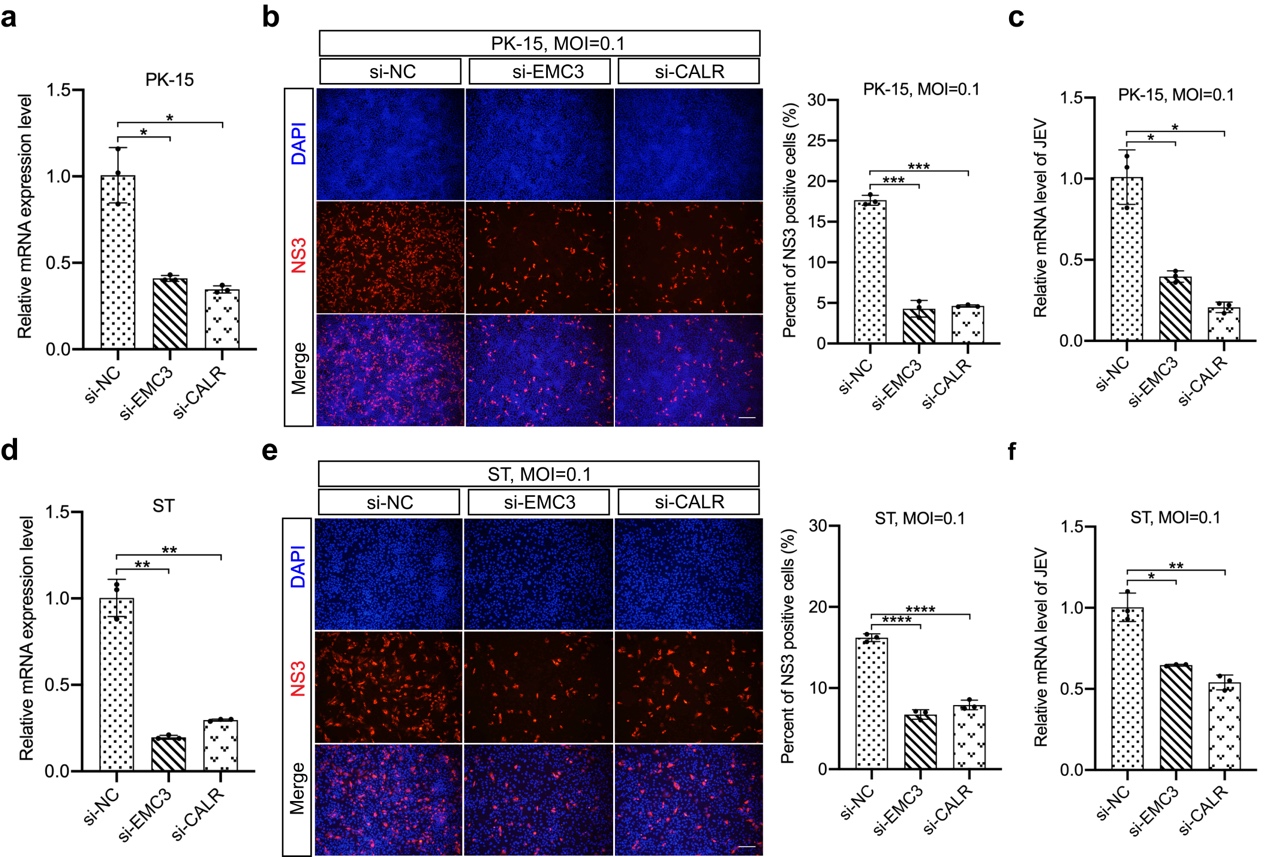
**

**Supplementary Fig. 17 Knockdown of EMC3 or CALR led to significant inhibition of JEV replication at an MOI of 0.1 in PK-15 or ST cells. a**, RT-qPCR assay for determination of relative mRNA level in a *EMC3* or *CALR* knockdown cells as compared with control PK-15 cells. **b**, Detection of NS3 protein by Immunofluorescence. The proportion of NS3 positive cells was significantly decrease in a *EMC3* or *CALR* knockdown cells as compared with control PK-15 cells. Left: representative pictures. Right: quantification of NS3 positive cells. **c**, RT-qPCR assay for determination of relative mRNA level of JEV *C* gene in a *EMC3* or *CALR* knockdown cells following infection with JEV at an MOI of 0.1 (PK-15 cells). *GAPDH* was used as a reference gene. **d**, RT-qPCR assay for determination of relative mRNA level in a *EMC3* or *CALR* knockdown cells as compared with control ST cells. **e**, Detection of NS3 protein by Immunofluorescence. The proportion of NS3 positive cells was significantly decreased in a *EMC3* or *CALR* knockdown cells as compared with control ST cells. Left: representative pictures. Right: quantification of NS3 positive cells. **f**, RT-qPCR assay for determination of relative mRNA level of JEV *C* gene in a *EMC3* or *CALR* knockdown cells following infection with JEV at an MOI of 0.1 (ST cells). *GAPDH* was used as a reference gene. si-NC: negative control siRNA; si-#(target): siRNA (knockdown cells); MOI: multiplicity of infection; DAPI: 4′, 6-diamidino-2-phenylindole; ST: a normal Sus scrofa testis-derived cell line; Quantitative analysis of Immunofluorescence (**b** and **e**) was used by Image J software. Scale bar, 200 μm. Data are represented as means ± S.D.; n = 3 (**a, b, c, d, e, f**). * *P* < 0.05; ** *P* < 0.01; *** *P* < 0.001; **** *P* < 0.0001. *P*-values were determined by two-sided Student’s t-test. Source data are provided as a Source Data file.


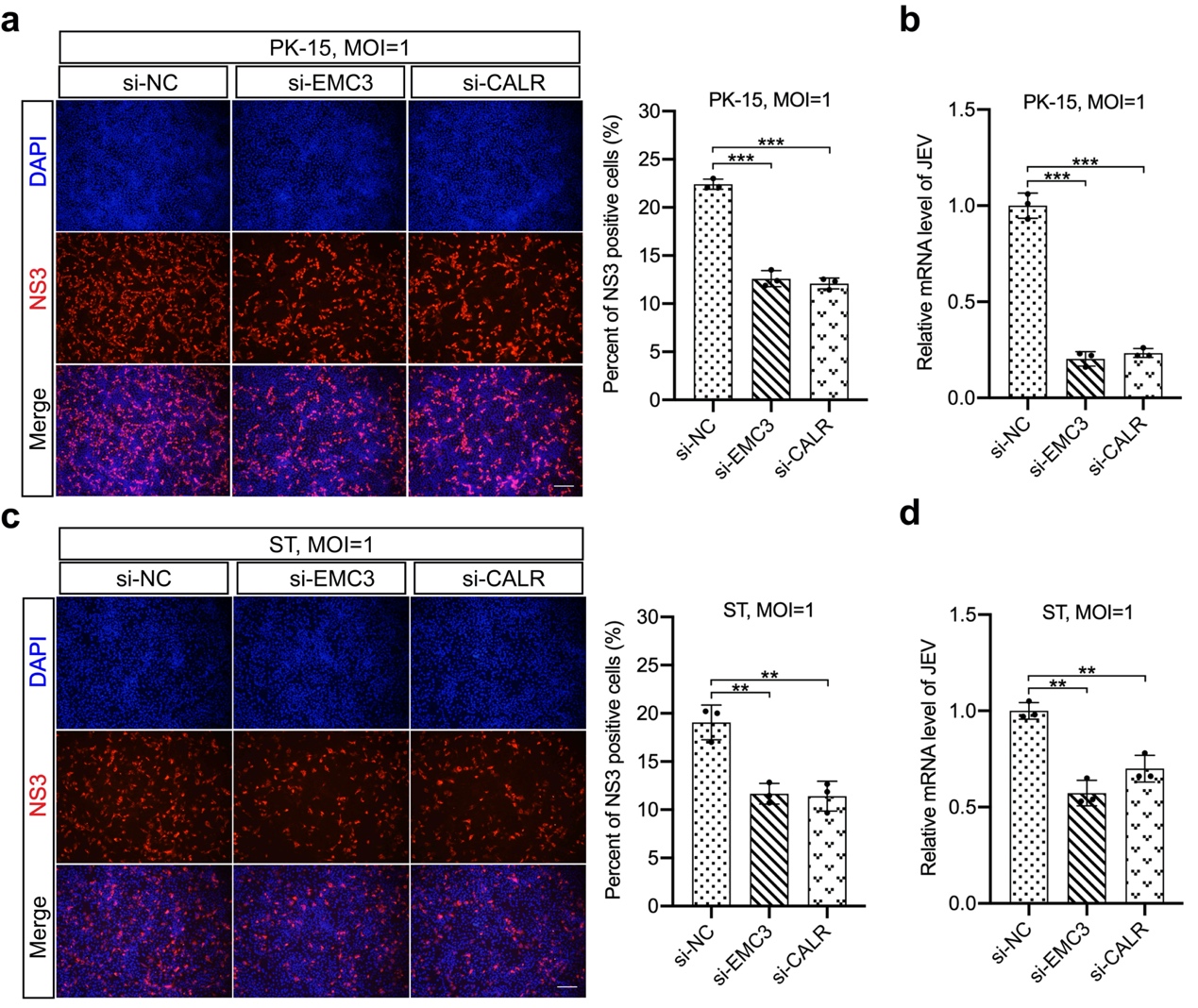


**Supplementary Fig. 18 Knockdown of EMC3 or CALR led to significant inhibition of JEV replication at an MOI of 1 in PK-15 or ST cells. a**, Detection of NS3 protein by Immunofluorescence. The proportion of NS3 positive cells was significantly decreased in a *EMC3* or *CALR* knockdown cells as compared with control PK-15 cells. Left: representative pictures. Right: quantification of NS3 positive cells. **b**, RT-qPCR assay for determination of relative mRNA level of JEV *C* gene in a *EMC3* or *CALR* knockdown cells following infection with JEV at an MOI of 1 (PK-15 cells). *GAPDH* was used as a reference gene. **c**, Detection of NS3 protein by Immunofluorescence. The proportion of NS3 positive cells was significantly decreased in a *EMC3* or *CALR* knockdown cells as compared with control ST cells. Left: representative pictures. Right: quantification of NS3 positive cells. **d**, RT-qPCR assay for determination of relative mRNA level of JEV *C* gene in a *EMC3* or *CALR* knockdown cells following infection with JEV at an MOI of 1 (ST cells). *GAPDH* was used as a reference gene. si-NC: negative control siRNA; si-#(target): siRNA (knockdown cells); MOI: multiplicity of infection; DAPI: 4′, 6-diamidino-2-phenylindole; ST: a normal Sus scrofa testis-derived cell line; Quantitative analysis of Immunofluorescence (**a** and **c**) was used by Image J software. Scale bar, 200 μm. Data are represented as means ± S.D.; n = 3 (**a, b, c, d**). ** *P* < 0.01; *** *P* < 0.001. *P*-values were determined by two-sided Student’s t-test. Source data are provided as a Source Data file.


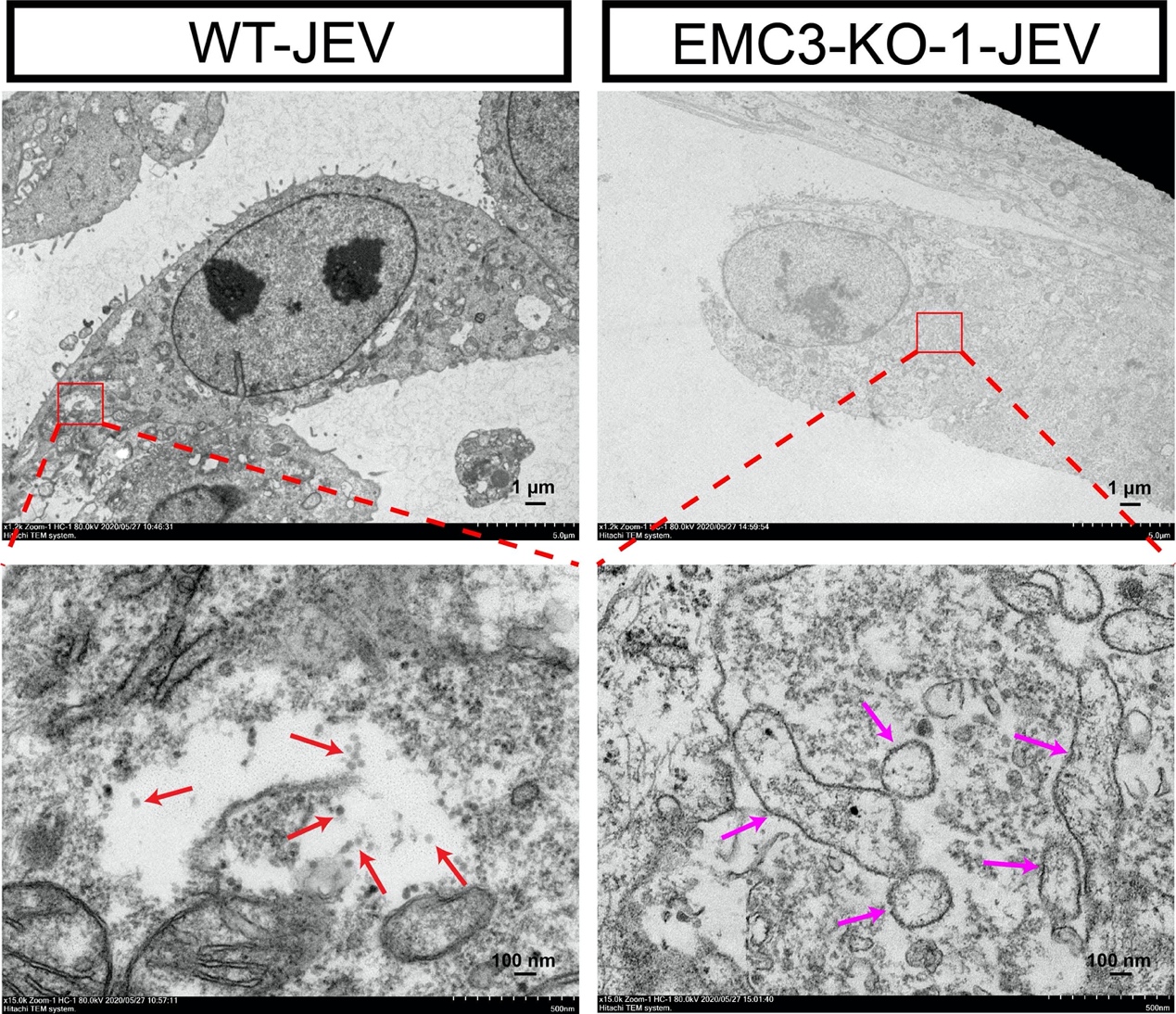


**Supplementary Fig. 19** **Observation of virus particle assembly and ER morphology in EMC3-deficient cells by negative-staining electron microscopy**. Numerous virus-like particles were present in WT cells (Red arrow). The pink arrow indicates EMC3-deficient cells displayed dramatic changes in ER morphology after JEV infection. Scale bar, 1 µm or 100 nm. Notes: The field of view of the microscope (Scale bar = 100 nm) is different from Fig. 5k.


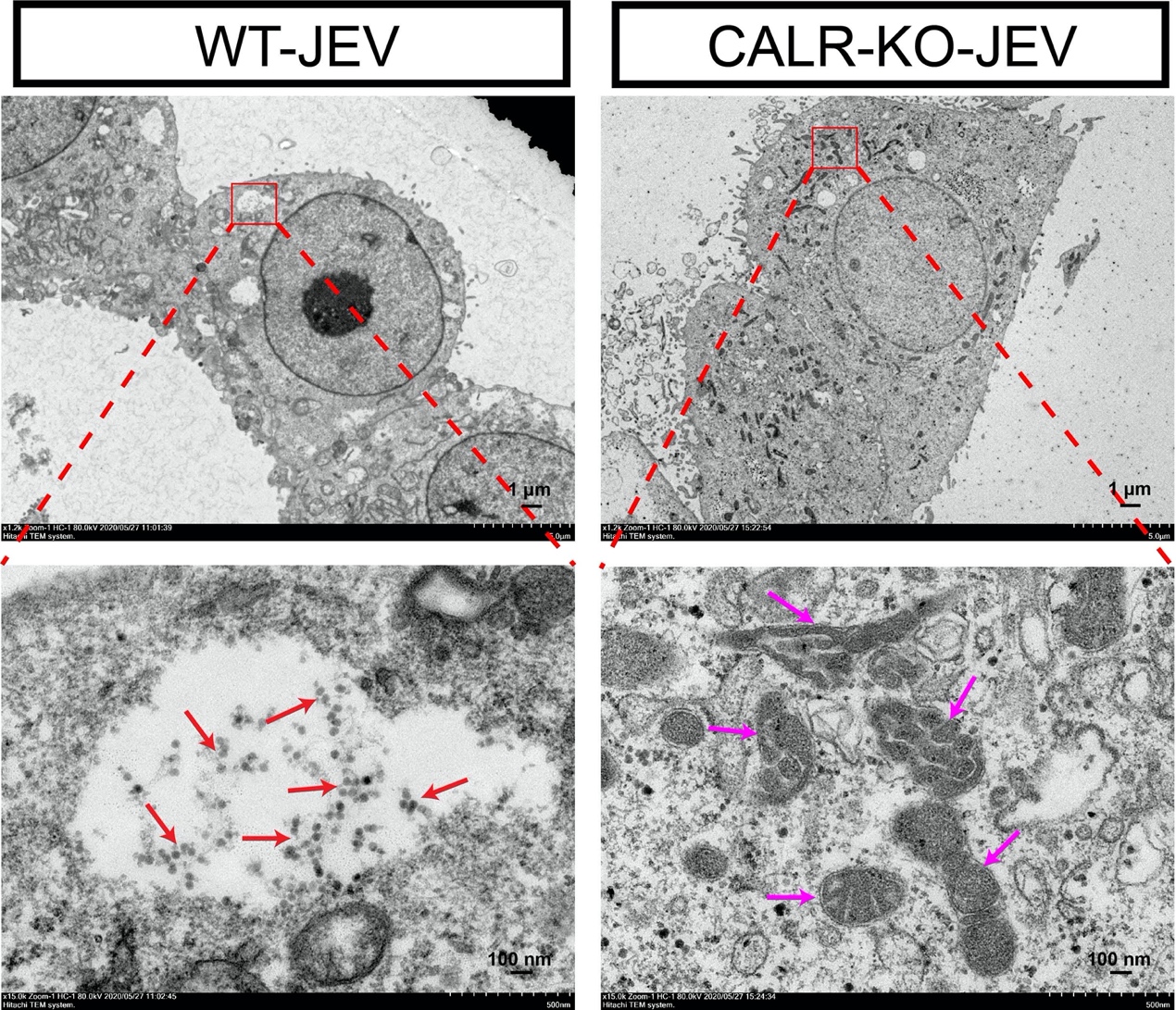


**Supplementary Fig. 20 Observation of virus particle assembly and mitochondrial morphology in CALR-deficient cells by negative-staining electron microscopy**. Numerous scattered virus-like particles were present in WT cells (Red arrow). The pink arrow indicates CALR-deficient cells displayed dramatic changes in mitochondrial morphology after JEV infection. Scale bar, 1 µm or 100 nm. Notes: The field of view of the microscope (Scale bar = 100 nm) is different from Fig. 6j.
