# Supplementary material for: CRISPR screening of porcine sgRNA library identifies host factors associated with Japanese encephalitis virus replication
Source: Nat Commun. 2020 Oct 14;11:5178. doi: 10.1038/s41467-020-18936-1 (PMC7560704; doi:10.1038/s41467-020-18936-1)
Supplement: Supplementary file 2 — Description of Additional Supplementary Files [file 41467_2020_18936_MOESM2_ESM.doc]

**File Name: Supplementary Data 1
Description:** Feature of the porcine genome-scale CRISPR/Cas9 knockout library.

**File Name: Supplementary Data 2**

**Description:** Sequencing results of sgRNAs targeting sequences in CRISPR knockout lentivirus plasmid pools and sorted mutant cell populations containing the entire sgRNA library.

**File Name: Supplementary Data 3**

**Description:** sgRNAs targeting sequences in the third or fourth rounds of JEV screens after challenge.

**File Name: Supplementary Data 4**

**Description:** Primer pairs and sgRNAs targeting sequences used in this study.
